# Relative Fat Mass as an estimator of whole-body fat percentage among children and adolescents: A cross-sectional study using NHANES

**DOI:** 10.1038/s41598-019-51701-z

**Published:** 2019-10-24

**Authors:** Orison O. Woolcott, Richard N. Bergman

**Affiliations:** Sports Spectacular Diabetes and Obesity Wellness and Research Center, Cedars-Sinai Medical Center, Los Angeles, CA 90048 USA

**Keywords:** Obesity, Paediatric research

## Abstract

We evaluated the ability of the Relative Fat Mass (RFM) to estimate whole-body fat percentage among children and adolescents who participated in the National Health and Nutrition Examination Survey from 1999 through 2006 (n = 10,390). The RFM equation for adults (64 − (20 × height/waist circumference) + (12 × sex)) may be used for adolescents 15 to 19 years of age. For children and adolescents 8 to 14 years of age, we suggest a modified RFM equation, named as the RFMp (RFM pediatric): 74 − (22 × height/waist circumference) + (5 × sex). In both equations, sex equals 0 for boys and 1 for girls. RFMp was more accurate than BMI to estimate whole-body fat percentage (measured by dual energy X-ray absorptiometry, DXA) among girls (percentage of estimates that were <20% of measured body fat percentage, 88.2% vs. 85.7%; P = 0.027) and boys 8 to 14 years of age (83.4% vs. 71.0%; P < 0.001). RFM was more accurate than BMI among boys 15 to 19 years of age (82.3% vs. 73.9%; P < 0.001) but slightly less accurate among girls (89.0% vs. 92.6%; P = 0.002). Compared with BMI-for-age percentiles, RFMp had lower misclassification error of overweight or obesity (defined as a DXA-measured body fat percentage at the 85^th^ percentile or higher) among boys 8 to 14 years of age (6.5% vs. 7.9%; P = 0.018) but not girls (RFMp: 8.2%; BMI-for-age: 7.9%; P = 0.681). Misclassification error of overweight or obesity was similar for RFM and BMI-for-age percentiles among girls (RFM: 8.0%; BMI-for-age: 6.6%; P = 0.076) and boys 15 to 19 years of age (RFM: 6.9%; BMI-for-age: 7.8%; P = 0.11). RFMp for children and adolescents 8 to 14 years of age and RFM for adolescents 15 to 19 years of age were useful to estimate whole-body fat percentage and diagnose body fat-defined overweight or obesity.

## Introduction

Latest reports from the Centers for Disease Control and Prevention indicate that the prevalence of obesity among children in the United States has remained unchanged in the last years but continues to increase among adolescents^1^. Body mass index (BMI) is a useful tool in pediatrics^1,2^. The use of age- and sex-specific BMI percentiles is the most recommended approach to assess body fatness among girls and boys between 2 and 19 years of age^3^. An acknowledged limitation of BMI is that it does not distinguish between fat mass and non-fat mass^4^. This limitation also extends to the pediatric population^5^. In fact, the rates of misclassification of body fat-defined obesity for BMI percentiles are overall high among girls compared with reference methods due to its lower sensitivity^6,7^. Another limitation of the utility of BMI in adolescents is that body weight is not proportional to height squared^8^, herein BMI requires adjustment for age to better classify overweight status in this population^1,2^.

High body fat percentage is associated with mortality in adults^9–11^. Childhood obesity, as defined by age-sex specific BMI percentiles, is associated with adult morbidity and mortality^12–14^. Early in life, high body fat percentage is associated with cardiovascular risk factors in children and adolescents^15^. Thus, given the strong evidence of an association between increased morbimortality and body fatness in children and adolescents^12–15^, it is important to have accurate estimates of body fat percentage.

Quadratic regression equations, based on the novel tri-ponderal mass index (TMI, body mass divided by the height cubed), have recently been proposed as low-cost and simpler alternatives to predict and estimate body fat percentage among European-American boys and girls^16^. More recently, we have proposed a new anthropometric equation named as the Relative Fat Mass (RFM), which is based on height/waist circumference ratio, as a more accurate method than BMI to estimate whole-body fat percentage among adult individuals^17^. Whether RFM is useful to assess body fatness among children and adolescents, remains unknown.

Height/waist ratio is the reciprocal of the widely used waist-to-height ratio (WHtR). Previous studies have shown that WHtR has a good linear relationship with BMI Z-scores in both girls and boys 2 to 7 years of age^18^. However, it appears that the association of body fat percentage with WHtR is not stronger than that with BMI in this population^19–21^. Conversely, in older children and adolescents, WHtR appears to have a stronger association with whole body adiposity than BMI does^22,23^. There is also evidence of better screening of whole body adiposity with waist circumference than that with BMI in boys 7 to 17 years of age^24^. However, the generalizability of previous studies are limited by the relatively small number of study participants^19,22,24^ and some studies did not use accepted reference methods to assess body fat percentage^20,22,23^. In a larger study, WHtR Z-scores have been shown to have a stronger agreement with body fat percentage than BMI did in girls and boys 8 to 19 years of age^25^. Equations based on WHtR to estimate body fat percentage have been suggested for children and adolescents 6 to 14 years of age^26^, but not for older adolescents. Whether equations based on waist circumference and height are better than BMI to estimate body adiposity in the pediatric population, remains unknown.

The main aim of the present study was to compare the ability of BMI, TMI and RFM to estimate whole-body fat percentage among children and adolescents. We also aimed to compare their clinical utility to discriminate body-fat defined overweight or obesity in the pediatric population.

## Results

### Study population

The final sample for analyses included 10,390 girls and boys. Characteristics of the study participants are shown in Table 1. Mean body fat percentage measured by dual energy X-ray absorptiometry (DXA) was 32.2 ± 0.3% and 27.3 ± 0.2% in girls and boys 8 to 14 years of age, respectively, and 34.4 ± 0.2% and 22.8 ± 0.2% in girls and boys 15 to 19 years of age, respectively.

**Table 1:** Characteristics of children and adolescents included in the study.

	8 to 14 years of age		15 to 19 years of age	
	Girls	Boys	Girls	Boys
*N*	2,383	3,085	2,110	2,812
Age, yr	11.1 ± 0.1	11.0 ± 0.1	17.1 ± 0.1	16.9 ± 0.1
**BMI-for-age-sex percentile** ^*^				
*Under weight (*<*5*^*th*^*), %*	3.0 ± 0.5	3.8 ± 0.5	3.3 ± 0.6	4.0 ± 0.6
*Healthy weight (≥5* ^*th*^ *to <85* ^*th*^ *), %*	62.0 ± 1.7	62.0 ± 1.3	65.9 ± 1.5	62.4 ± 1.3
*Overweight (≥85* ^*th*^ *to <95* ^*th*^ *), %*	16.6 ± 1.0	16.2 ± 0.9	15.9 ± 1.0	15.3 ± 0.9
*Obesity (≥95* ^*th*^ *) %*	18.5 ± 1.4	18.0 ± 1.2	15.0 ± 1.2	18.3 ± 1.2
*Overweight or obesity (≥85* ^*th*^ *), %*	35.0 ± 1.f8	34.2 ± 1.5	30.9 ± 1.4	33.6 ± 1.4
**Anthropometry**				
Body weight, kg	47.2 ± 0.6	46.4 ± 0.4	64.4 ± 0.6	75.1 ± 0.5
Height, cm	149.2 ± 0.3	149.8 ± 0.3	162.9 ± 0.1	175.7 ± 0.2
BMI, kg/m^2^	20.7 ± 0.2	20.1 ± 0.1	24.2 ± 0.2	24.2 ± 0.2
Waist circumference, cm	72.7 ± 0.5	71.5 ± 0.4	82.4 ± 0.5	84.5 ± 0.4
Waist-to-height ratio	0.49 ± 0.00	0.48 ± 0.00	0.51 ± 0.00	0.48 ± 0.00
Whole-body fat mass, kg	16.0 ± 0.3	13.4 ± 0.2	23.2 ± 0.4	18.3 ± 0.3
Whole-body fat free mass, kg	30.1 ± 0.3	32.0 ± 0.2	39.7 ± 0.2	55.0 ± 0.3
Whole-body fat percentage	32.2 ± 0.3	27.3 ± 0.2	34.4 ± 0.2	22.8 ± 0.2
Trunk fat percentage	28.1 ± 0.3	23.2 ± 0.3	30.9 ± 0.3	20.8 ± 0.3

Values represent pooled weighted mean estimates (or percentages, as indicated) ± standard errors.

BMI, body mass index (weight in kilograms divided by the square of the height in meters). *Percentages may not total 100 due to rounding.

### Derivation of equations for pediatric populations

As shown in Supplementary Fig. 1, body weight, height, waist circumference, and body composition substantially differed across age categories. The RFM equation that we previously developed for adults^17^ may be used to estimate whole-body fat percentage among adolescents 15 to 19 years of age. For younger children and adolescents (8 to 14 years of age), we developed a modified RFM equation named as the RFM pediatric (RFMp). RFM equations for pediatric populations are as follows:

1 $${\rm{Equation}}\,{\rm{for}}\,{\rm{girls}}\,8\,{\rm{to}}\,14\,{\rm{years}}\,{\rm{of}}\,{\rm{age}}:79-(22\times {\rm{height}}/{\rm{waist}})$$

2 $${\rm{Equation}}\,{\rm{for}}\,{\rm{boys}}\,8\,{\rm{to}}\,14\,{\rm{years}}\,{\rm{of}}\,{\rm{age}}:74-(22\times {\rm{height}}/{\rm{waist}})$$

or

3 $${\rm{RFMp}}\,{\rm{for}}\,{\rm{girls}}\,{\rm{and}}\,{\rm{boys}}\,8\,{\rm{to}}\,14\,{\rm{years}}\,{\rm{of}}\,{\rm{age}}:74-(22\times ({\rm{height}}/{\rm{waist}}))+(5\times {\rm{sex}})$$

4 $${\rm{RFM}}\,{\rm{for}}\,{\rm{girls}}\,{\rm{and}}\,{\rm{boys}}\,15\,{\rm{to}}\,19\,{\rm{years}}\,{\rm{of}}\,{\rm{age}}:64-(20\times {\rm{height}}/{\rm{waist}})+(12\times {\rm{sex}})$$

In all equations, height and waist (circumference) are in the same units. U.S./English units or Metric units may be used. In (3) and (4), sex equals 0 for boys and 1 for girls. For comparison purposes, we also derived equations based on BMI, TMI, and WHtR using the same NHANES dataset. Note that for a fair comparison among indices, we derived sex-specific equations for two groups: 1) for children and adolescents 8 to 14 years of age and 2) for adolescents 15 to 19 years of age. All raw (non-rounded) equations are shown in Supplementary Table 1. Since the relationship of BMI and TMI with body fat percentage is nonlinear^16^, quadratic equations were developed for BMI and TMI. Plots of RFMp and RFM as a function of age are shown in Supplementary Fig. 2A,C for girls and boys 8 to 14 years of age and in Supplementary Fig. 2B,D for girls and boys 15 to 19 years of age. In younger boys, there is a progressive subtle decrease in DXA-measured body fat percentage as age increases, which is paralleled by RFMp but not BMI (Supplementary Fig. 2C).

### Prediction of body fat percentage in children and adolescents 8 to 14 years of age

**Figure 1:**
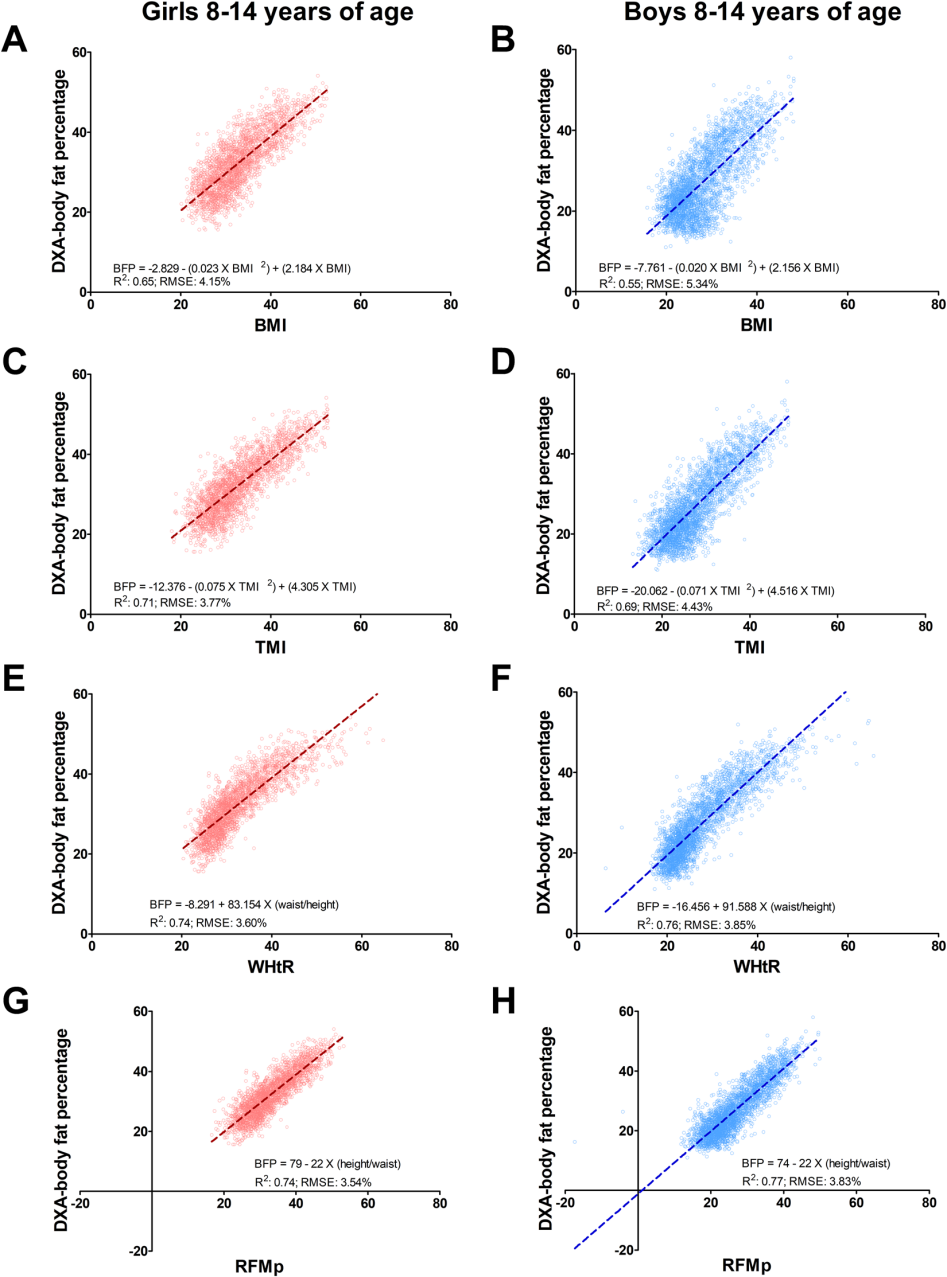
Linear relationship between DXA-measured and estimated body fat percentage among children and adolescents 8 to 14 years of age. BFP, body fat percentage; BMI, body mass index (weight/height^2^); RFMp, Relative Fat Mass pediatric, which is based on height/waist circumference. R^2^, coefficient of determination; RMSE, root mean squared error; TMI, tri-ponderal mass index (weight/height^3^); WHtR, waist-to-height ratio. Data plots correspond to DXA imputation 1.

Compared with BMI, RFMp showed a better linear relationship with DXA whole-body fat percentage among girls (RFMp: R^2^, 0.74; 95% confidence interval (CI), 0.72−0.77; root mean squared error (RMSE): 3.83%; 95% CI, 3.70%-3.97%; BMI: R^2^, 0.65; 95% CI, 0.62−0.68; RMSE: 4.15%; 95% CI, 4.00%-4.29%) and boys (RFMp: R^2^, 0.77; 95% CI, 0.75−0.79; RMSE: 3.83%; 95% CI, 3.70%-3.97%; BMI: R^2^, 0.55; 95% CI, 0.52−0.57; RMSE: 5.34%; 95% CI, 5.19%-5.49%). RFMp also showed a better linear relationship with DXA whole-body fat percentage compared with TMI among girls (TMI: R^2^, 0.71; 95% CI, 0.69−0.73; RMSE: 3.77%; 95% CI, 3.64%-3.90%). RFMp was a better predictor than TMI among boys (TMI: R^2^, 0.69; 95% CI, 0.66−0.71; RMSE: 4.43%; 95% CI, 4.29%-4.56%) (Fig. 1). Body fat linear prediction showed some variability with age, regardless the index used (Supplementary Table 2). RFMp was better than BMI in predicting body fat percentage across ethnic groups in girls (Supplementary Fig. 3A–F) and boys (Supplementary Fig. 4A–F).

RFMp and WHtR showed similar predicting ability for whole-body fat percentage among girls (WHtR: R^2^, 0.74; 95% CI 0.72−0.76; RMSE: 3.60%; 95% CI, 3.48–3.71%) and boys (WHtR: R^2^, 0.76; 95% CI, 0.75−0.78; RMSE: 3.85%; 95% CI 3.72–3.99%). However, RFMp was a better predictor than WHtR among African-American girls (RFMp: R^2^, 0.76; 95% CI 0.72−0.79; RMSE: 3.80%; 95% CI, 3.61–3.99%; WHtR: R^2^, 0.73; 95% CI 0.69−0.77; RMSE: 4.00%; 95% CI, 3.78–4.21%).

### Prediction of body fat percentage in adolescents 15 to 19 years of age

**Figure 2:**
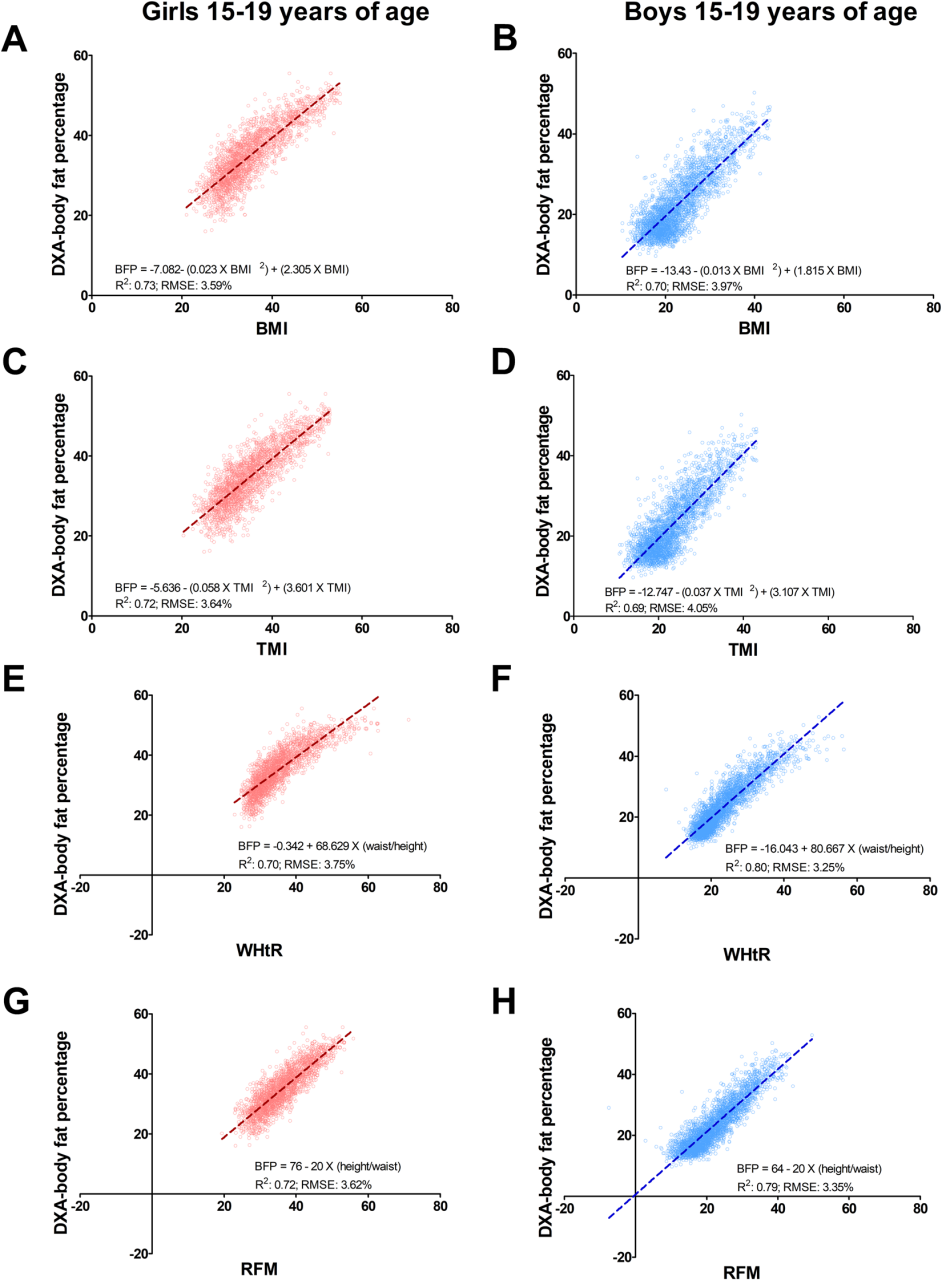
Linear relationship between DXA-measured and estimated body fat percentage among adolescents 15 to 19 years of age. BFP, body fat percentage; BMI, body mass index; RFM, Relative Fat Mass. R^2^, coefficient of determination; RMSE, root mean squared error; TMI, tri-ponderal mass index; WHtR, waist-to-height ratio. Data plots correspond to DXA imputation 1.

Compared with BMI, RFM equation for adults showed a better linear relationship with DXA whole-body fat percentage among boys (RFM: R^2^, 0.79; 95% CI, 0.76−0.81; RMSE: 3.35%; 95% CI, 3.22–3.49%; BMI: R^2^, 0.70; 95% CI, 0.67−0.73; RMSE: 3.97%; 95% CI, 3.83–4.12%) but not girls (RFM: R^2^, 0.72; 95% CI, 0.70−0.75; RMSE: 3.45%; 95% CI, 3.26–3.63%; BMI: R^2^, 0.73; 95% CI, 0.70−0.75; RMSE: 3.59%; 95% CI, 3.45–3.73%). RFM also showed a better linear relationship with DXA whole-body fat percentage compared with TMI among boys (TMI: R^2^, 0.69; 95% CI, 0.66−0.72; RMSE: 4.05%; 95% CI, 3.90–4.19%) but not girls (TMI: R^2^, 0.72; 95% CI, 0.69−0.74; RMSE: 3.64%; 95% CI, 3.51–3.78%) (Fig. 2). All indices showed variable prediction with age (Supplementary Table 3). RFM was better than BMI in predicting body fat percentage across ethnic groups among boys (Supplementary Fig. 4G–L) but not girls (Supplementary Fig. 3G–L). Highest prediction of body fat percentage by RFM was found among African-American adolescents (Supplementary Figs 3L and 4L).

WHtR appeared to have slightly lower predicting ability than RFM among girls (WHtR: R^2^, 0.70; 95% CI 0.68−0.73; RMSE: 3.75%; 95% CI, 3.58–3.91%) but not boys (WHtR: R^2^, 0.80; 95% CI, 0.78−0.82; RMSE: 3.25%; 95% CI 3.12–3.39%). However, RFM was a better predictor than WHtR among African-American girls (RFM: R^2^, 0.80; 95% CI 0.77−0.83; RMSE: 3.57%; 95% CI, 3.35–3.80%; WHtR: R^2^, 0.75; 95% CI 0.72−0.79; RMSE: 3.96%; 95% CI, 3.70–4.21%).

### Agreement between DXA-measured and estimated whole-body fat percentage

Concordance analyses for RFMp and RFM linear equations were performed using their rounded and simplest expressions as indicated in (3) and (4) and compared with the raw quadratic equations for BMI and TMI (Supplementary Table 1). Among girls 8 to 14 years of age, the concordance correlation coefficients (ρc) were 0.85, 0.84 and 0.79 for RFMp, TMI and BMI equations, respectively. Among boys 8 to 14 years of age, RFMp appeared to show better agreement with DXA (ρc: 0.86) than did TMI equation (ρc: 0.80) or BMI (ρc: 0.70). Among girls 15 to 19 years of age, RFM, TMI and BMI equations showed good agreement with DXA. Concordance correlation coefficients were 0.83, 0.84 and 0.85, respectively. Among boys 15 to 19 years of age, RFM appeared to show better agreement with DXA (ρc: 0.86) than did TMI equation (ρc: 0.81) or BMI (ρc: 0.82). Overall, Bland-Altman plots showed good agreement between DXA-measured body fat percentage and RFMp-estimated body fat percentage among girls and boys 8 to 14 years of age of different ethnicities (Supplementary Fig. 5). Likewise, we found good agreement between DXA-measured body fat percentage and RFM-estimated body fat percentage among adolescents 15 to 19 years of age of different ethnicities (Supplementary Fig. 6).

### RFMp performance among children and adolescents 8 to 14 years of age

RFMp linear equation showed higher accuracy than BMI quadratic equation to estimate whole-body fat percentage among girls (RFMp: 88.2%; 95% CI, 86.5–89.9%: BMI: 85.7%; 95% CI, 83.7–87.6%; P = 0.027) and boys (RFMp: 83.4%; 95% CI, 81.5–85.4%; BMI: 71.0%; 95% CI, 68.8–73.3%; P < 0.001). TMI quadratic equation had similar accuracy to RFMp among girls (TMI: 88.7%; 95% CI, 87.3–90.0%;P = 0.521) but was less accurate than RFMp among boys (TMI: 77.3%; 95% CI, 75.1–79.4%; P < 0.001). As indicated by a smaller interquartile range of the difference between DXA-measured and estimated whole-body fat percentage, RFMp was also more precise than BMI among girls (RFMp: 5.00%; 95% CI, 4.73–5.28%; BMI: 5.71%; 95% CI, 5.31–6.12%) and boys (RFMp: 5.01%; 95% CI, 4.67–5.35%; BMI: 7.16%; 95% CI, 6.72–7.59%). Likewise, RFMp was more precise than TMI among girls (TMI: 5.21%; 95% CI, 4.87–5.55%) and boys (TMI: 5.98%; 95% CI, 5.60–6.35%) (Supplementary Table 4). Overall, RFMp was more accurate than BMI across ethnic groups (Supplementary Table 4). RFMp also showed higher accuracy than BMI across body fat ranges among boys (Supplementary Fig. 7E) and across higher quartiles among girls (Supplementary Fig. 7B), but accuracy was lower in leaner individuals (Supplementary Fig. 7). Accuracy of RFMp was also more consistent than that of BMI across age groups (Supplementary Fig. 8).

### RFM performance among adolescents 15 to 19 years of age

RFM linear equation showed higher accuracy than BMI quadratic equation among boys (RFM: 82.3%; 95% CI, 80.3–84.2% vs. BMI: 73.9%; 95% CI, 71.3–76.5%; P < 0.001) but was less accurate than BMI among girls (RFM: 89.0%; 95% CI, 86.7–91.2% vs. BMI: 92.6%; 95% CI, 91.1–94.1%; P = 0.002). RFM was less accurate than BMI among European-American girls (P = 0.002) and African-American girls (P = 0.015) but not Mexican-American girls (P = 0.384) (Supplementary Table 5). Conversely, RFM was more accurate than BMI across male ethnic groups (Supplementary Table 5) and across age groups among boys (Supplementary Fig. 8). TMI was less accurate than RFM among boys (TMI: 72.8%; 95% CI, 70.0–75.6%; P < 0.001) but more accurate than RFM among girls (TMI: 91.5%; 95% CI, 90.0–93.0%;P = 0.028), but only among European-American girls (RFM: 89.2%; 95% CI, 86.1–92.2%; TMI: 92.6%; 95% CI, 90.3–94.8%; P = 0.015). In fact, RFM was more accurate than TMI among African-American girls (RFM: 86.7%; 95% CI, 84.2–89.2%; TMI: 84.1%; 95% CI, 80.9–87.2%; P = 0.001) (Supplementary Table 5). Overall, RFM showed similar accuracy to BMI and TMI at higher body fat ranges but RFM accuracy was lower than BMI and TMI in leaner girls and higher in leaner boys (Supplementary Fig. 9B,E). RFM was also more precise than BMI among boys (4.51%; 95% CI, 4.21–4.80% vs. 5.09%; 95% CI, 4.79–5.39%) but not girls (4.64%; 95% CI, 4.35–4.93% vs. 4.78%; 95% CI, 4.52–5.05%) (Supplementary Table 5). Likewise, RFM was more precise than TMI among boys (TMI: 5.25%; 95% CI, 4.94–5.56%) but not girls (TMI: 4.76%; 95% CI, 4.43–5.08%).

### Overweight and obesity misclassification among children and adolescents 8 to 14 years of age

Analyses were performed with data from 5,395 children and adolescents 96 to 179 months old (2,355 girls). For TMI, overweight was defined as ≥17.0 kg/m^3^ and <19.8 kg/m^3^ for girls, and ≥16.2 kg/m^3^ and <19.2 kg/m^3^ for boys. Obesity was defined as a TMI ≥ 19.8 kg/m^3^ for girls and ≥19.2 kg/m^3^ for boys. For RFMp, overweight was defined as ≥40.0% and <44.0% for girls, and ≥34.5% and <39.3% for boys. Obesity was defined as an RFMp ≥ 44.0% for girls and ≥39.3% for boys. The aforementioned RFMp thresholds were arbitrarily chosen for comparison purposes only based on the commonly used 85^th^ and 95^th^ percentiles and are not intended to suggest RFMp cutoffs for the diagnosis of overweight and obesity.

RFMp showed similar false negative rate for overweight or obesity (defined as a DXA-measured body fat percentage ≥85^th^ percentile) compared with BMI-for-age percentiles among girls (RFMp: 26.7%; 95% CI, 20.6–32.8%; BMI-for-age: 25.8%; 95% CI, 18.9–32.8%; P = 0.783) and boys (RFMp: 21.8%; 95% CI, 17.0–26.6%; BMI-for-age: 25.8%; 95% CI, 19.1–32.6%; P = 0.208) (Table 2). RFMp showed lower false positive rate among boys (RFMp: 3.7%; 95% CI, 2.8–4.7%; BMI-for-age: 4.7%; 95% CI, 3.7–5.7%; P = 0.045), but not girls (RFMp: 5.0%; 95% CI, 3.7–6.2%; BMI-for-age: 4.8%; 95% CI, 3.2–6.3%; P = 0.742) (Table 2).

**Table 2:** Positive and negative cases of DXA-diagnosed overweight or obesity for RFMp and RFM among children and adolescents 96–239 months old (n = 10,260).

	Diagnosis by DXA n (%)		
**Diagnosis by RFMp** **(Girls and boys 8 to 14 years of age)**	**Negative**	**Positive**	**Total, n (%)**
Negative	4,256 (78.9)	199 (3.7)	4,455 (82.6)
Positive	253 (4.7)	687 (12.7)	940 (17.4)
Total	4,509 (83.6)	886 (16.4)	5,395 (100)
**Diagnosis by RFM** **(Girls and boys 15 to 19 years of age)**	**Negative**	**Positive**	**Total**
Negative	3,846 (79.1)	200 (4.1)	4,046 (83.2)
Positive	213 (4.4)	606 (12.5)	819 (16.8)
Total	4,050 (83.4)	806 (16.6)	4,865 (100)

Overweight or obesity was defined as a DXA body fat percentage ≥85^th^ percentile. DXA, dual energy X-ray absorptiometry. DXA data estimated from Imputation 1.

**Figure 3:**
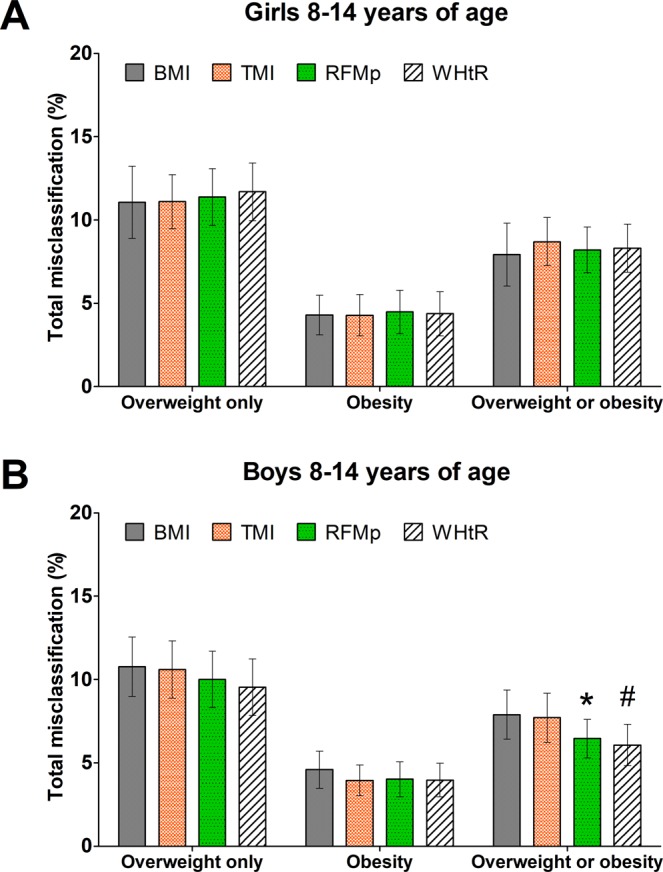
Comparison of total misclassification error rate of body adiposity between indices in children and adolescents 8 to 14 years of age. BMI, body mass index; RFMp, Relative Fat Mass pediatric; TMI, tri-ponderal mass index; WHtR, waist-to-height ratio. Bars show comparison of total misclassification of overweight only, obesity, and overweight or obesity diagnosed by DXA-whole-body fat percentage (≥85^th^ percentile and <95^th^ percentile for overweight and ≥95^th^ percentile for obesity). Error bars are 95% confidence intervals. *P < 0.01; #P < 0.05; compared with BMI.

RFMp and BMI-for-age percentiles had similar total misclassification error rates of overweight (girls: P = 0.685; boys: P = 0.322) and obesity (girls: P = 0.631; boys: P = 0.192) (Fig. 3). RFMp had lower misclassification error rate of overweight or obesity than BMI-for-age percentiles among boys (RFMp: 6.5%; 95% CI, 5.3–7.6%; BMI-for-age: 7.9%; 95% CI, 6.4–9.4%; P = 0.018) but not girls (RFMp: 8.2%; 95% CI, 6.8–9.6%; BMI-for-age: 7.9%; 95% CI, 6.0–9.8%; P = 0.681). RFMp and TMI had similar rates of misclassification of overweight (girls: P = 0.729; boys: P = 0.412) and obesity (girls: P = 0.657; boys: P = 0.887). Conversely, RFMp had lower misclassification error of overweight or obesity than TMI among boys (TMI: 7.7%; 95% CI, 6.2–9.2%; P = 0.016) but not girls (P = 0.444). WHtR had a slightly lower misclassification error rate of overweight or obesity than RFMp among boys (WHtR: 6.1%; 95% CI, 4.8–7.3%; P = 0.041) but not girls (WHtR: 8.3%; 95% CI, 6.9–9.7%; P = 0.785) (Fig. 3).

RFMp had lower total misclassification error of overweight or obesity than BMI-for-age percentiles among African-American girls (RFMp: 8.2%; 95% CI, 6.1–10.3%; BMI-for-age: 11.3%; 95% CI, 8.8–13.8%; P < 0.001) and African-American boys (RFMp: 6.5%; 95% CI, 4.7–8.2%; BMI-for-age: 8.7%; 95% CI, 6.6–10.7%; P = 0.009) (Supplementary Fig. 10). Among African-Americans girls and boys together, RFMp had lower total misclassification error than BMI-for-age percentiles (RFMp: 7.2%; 95% CI, 5.8–8.6%; BMI-for-age: 9.8%; 95% CI, 8.2–11.3%; P < 0.001).

When using thresholds based on the highest Youden’s index, RFMp (threshold for girls and boys: 37.6% and 32.8%, respectively) and BMI-for-age had similar rates of misclassification of overweight or obesity among girls (RFMp: 11.9%; 95% CI, 10.1–13.7%; BMI-for-age: 11.6%; 95% CI, 9.5–13.7%; P = 0.796) and boys (RFMp: 8.2%; 95% CI, 6.9–9.6%; BMI-for-age: 9.3%; 95% CI, 7.9–10.7%; P = 0.109). RFMp had lower misclassification error of overweight or obesity than BMI-for age among African-American girls (RFMp: 12.5%; 95% CI, 10.3–14.7%; BMI-for-age: 17.6%; 95% CI, 14.7–20.4%; P < 0.001) and African-American boys (RFMp: 6.7%; 95% CI, 4.9–8.5%; BMI-for-age: 12.6%; 95% CI, 10.0–15.3%; P < 0.001). Overall, RFMp showed lower false negative rates and lower false positive rates for overweight and obesity compared with BMI among girls and boys (Supplementary Table 6).

### Overweight and obesity misclassification among adolescents 15 to 19 years of age

Analyses were performed with data from 4,865 adolescents 180 to 239 months old (2,080 girls). For TMI, overweight was defined as ≥18.2 kg/m^3^ and <22.3 kg/m^3^ for girls, and ≥16.9 kg/m^3^ and <20.1 kg/m^3^ for boys. Obesity was defined as a TMI ≥ 22.3 kg/m^3^ for girls and ≥20.1 kg/m^3^ for boys. For RFM, overweight was defined as ≥42.3% and <46.2% for girls, and ≥28.6% and <32.9% for boys. Obesity was defined as an RFM ≥ 46.2% for girls and ≥32.9% for boys. The aforementioned RFM thresholds are not intended to suggest cutoffs for the diagnosis of overweight and obesity in this pediatric population.

RFM showed similar false negative rate for overweight or obesity compared with BMI-for-age percentiles among girls (RFM: 26.8%; 95% CI, 20.9–32.8%; BMI-for-age: 21.8%; 95% CI, 15.5–28.1%; P = 0.165) and boys (RFM: 22.9%; 95% CI, 16.9–28.9%; BMI-for-age: 24.3%; 95% CI, 18.1–30.4%; P = 0.564) (Table 2). Likewise, RFM showed similar false positive rate among girls (RFM: 4.7%; 95% CI, 3.5–5.9%; BMI-for-age: 4.0%; 95% CI, 2.8–5.1%; P = 0.253), and boys (RFM: 4.2%; 95% CI, 3.0–5.3%; BMI-for-age: 4.9%; 95% CI, 3.6–6.1%; P = 0.145) (Table 2).

**Figure 4:**
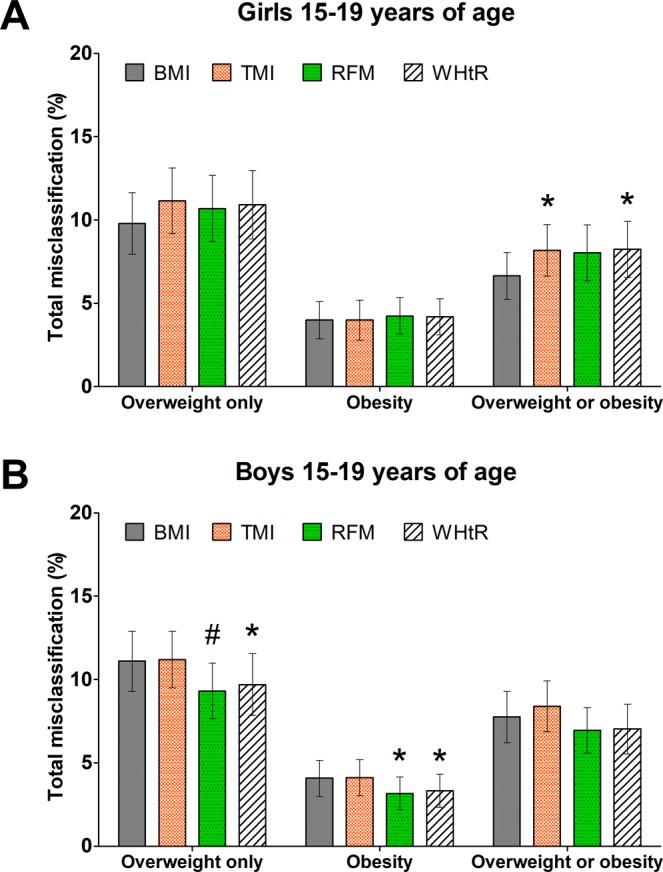
Comparison of total misclassification error rate of body adiposity between indices in adolescents 15 to 19 years of age. BMI, body mass index; RFM, Relative Fat Mass; TMI, tri-ponderal mass index; WHtR, waist-to-height ratio. Bars show comparison of total misclassification of overweight only, obesity, and overweight or obesity diagnosed by DXA-whole-body fat percentage (≥85^th^ percentile and <95^th^ percentile for overweight and ≥95^th^ percentile for obesity). Error bars are 95% confidence intervals. *P < 0.05; #P < 0.01; compared with BMI.

RFM had lower total misclassification error rate of overweight than BMI-for-age percentiles among boys (P = 0.006) but not girls (P = 0.271). Likewise, RFM had lower total misclassification error rate of obesity than BMI-for-age percentiles among boys (P = 0.012) but not girls (P = 0.456) (Fig. 4). RFM and BMI-for-age percentiles had similar total misclassification error of overweight or obesity among girls (RFM: 8.0%; 95% CI, 6.4–9.7%; BMI-for-age: 6.6%; 95% CI, 5.2–8.0%; P = 0.076) and boys (RFM: 6.9%; 95% CI, 5.6–8.3%; BMI-for-age: 7.8%; 95% CI, 6.2–9.3%; P = 0.11). RFM had lower total misclassification error rate of overweight than TMI among boys (P = 0.006) but not girls (P = 0.526). Likewise, RFM had lower total misclassification error rate of obesity than TMI among boys (P = 0.005) but not girls (P = 0.557). RFM also had lower misclassification error of overweight or obesity than TMI among boys (TMI: 8.4%; 95% CI, 6.9–9.9%; P = 0.015) but not girls (TMI: 8.2%; 95% CI, 6.6–9.7%; P = 0.789). RFM and WHtR had similar rates of misclassification of overweight or obesity among girls (WHtR: 8.2%; 95% CI, 6.5–9.9%; P = 0.30) and boys (WHtR: 7.0%; 95% CI, 5.5–8.5%; P = 0.781) (Fig. 4).

RFM had lower total misclassification error of overweight or obesity than BMI-for-age percentiles among African-American boys (RFM: 6.3%; 95% CI, 4.5–8.2%; BMI-for-age: 8.0%; 95% CI, 6.3–9.8%; P = 0.024) but not girls (RFM: 9.2%; 95% CI, 7.1–11.3%; BMI-for-age: 10.4%; 95% CI, 7.6–13.1%; P = 0.378) (Supplementary Fig. 11). Among African-Americans girls and boys together, RFM had lower total misclassification error than BMI-for-age percentiles (RFM: 7.6%; 95% CI, 6.1–9.0%; BMI-for-age: 9.0%; 95% CI, 7.5–10.6%; P < 0.001).

When using thresholds based on the highest Youden’s index, RFM (threshold for girls and boys: 38.9% and 26.2%, respectively) had lower total misclassification error of overweight or obesity than BMI-for-age among boys (RFM: 10.1%; 95% CI, 8.5–11.8%; BMI-for-age: 12.7%; 95% CI, 11.1–14.2%; P < 0.001) and higher misclassification error among girls (RFM: 13.2%; 95% CI, 11.2–15.2%; BMI-for-age: 9.6%; 95% CI, 8.1–11.0%; P < 0.001). RFM had lower misclassification error of overweight or obesity than BMI-for age among African-American boys (RFM: 7.8%; 95% CI, 6.1–9.4%; BMI-for-age: 12.6%; 95% CI, 10.2–15.0%; P < 0.001) but not girls (RFM: 16.4%; 95% CI, 13.4–19.4%; BMI-for-age: 17.5%; 95% CI, 14.5–20.5%; P = 0.181). Compared with BMI, RFM showed lower false negative rates and lower false positive rates for overweight among boys and lower false positive rates for obesity among boys (Supplementary Table 6).

### Diagnostic accuracy of overweight or obesity

Among children and adolescents 8 to 14 years of age, compared with BMI, RFMp had a better diagnostic accuracy of overweight or obesity among girls (C-statistic: 0.95; 95% CI, 0.94−0.96 vs. 0.93; 95% CI, 0.91−0.95; P = 0.001) and boys (C-statistic: 0.97; 95% CI, 0.96−0.98 vs. 0.95; 95% CI, 0.94−0.96; P < 0.001). Among adolescents 15 to 19 years of age, compared with BMI, RFM had a better diagnostic accuracy of overweight or obesity among boys (C-statistic: 0.97; 95% CI, 0.96−0.98 vs. 0.95; 95% CI, 0.93−0.96; P < 0.001) but not girls (C-statistic: 0.96; 95% CI, 0.95−0.97 vs. 0.96; 95% CI, 0.95−0.97; P = 0.588).

Among children and adolescents 8 to 19 years of age, compared with BMI, height/waist ratio (the basis of both RFMp and RFM equations) showed better diagnostic accuracy of overweight or obesity among girls (C-statistic: 0.95; 95% CI, 0.94−0.96 vs. 0.92; 95% CI, 0.90−0.94; P < 0.001) and boys (C-statistic: 0.97; 95% CI, 0.96−0.97 vs. 0.93; 0.92−0.93; P < 0.001).

### Association of RFMp and RFM with biomarkers for cardiometabolic disease

Among girls 8 to 14 years of age, RFMp showed the strongest association with log-insulin (r = 0.59; P < 0.001) and log-(triacylglycerol-HDLc-ratio) (r = 0.31; P < 0.001) compared with other biomarkers. Among boys 8 to 14 years of age, RFMp also showed the strongest association with log-insulin (r = 0.63; P < 0.001) and log-(triacylglycerol-HDLc-ratio) (r = 0.48; P < 0.001) (Supplementary Table 7). Among girls 15 to 19 years of age, RFM showed the strongest association with log-insulin (r = 0.48; P < 0.001) and log-HDLc (r = −0.34; P < 0.001). Among boys 15 to 19 years of age, RFM showed the strongest association with log-insulin (r = 0.59; P < 0.001) and log-(triacylglycerol-HDLc-ratio) (r = 0.45; P < 0.001) (Supplementary Table 8).

## Discussion

Numerous anthropometric equations have been proposed as alternatives to BMI to estimate whole-body fat percentage in adult individuals^27–36^. However, a major problem with previous equations is the lack of simplicity, showing limited utility for clinical and epidemiological purposes. Recently, we developed and validated the RFM as a more accurate method than BMI to estimate whole-body fat percentage among adult individuals^17^. In the present study, we wanted to evaluate the utility of RFM to assess body fatness in the pediatric population. We used a representative sample of the U.S. population who participated in the NHANES from 1999 through 2006, comprised of children and adolescents 8 to 19 years of age who had available information on DXA-assessed whole-body composition.

Our findings suggest that the modified RFM equation for adults, which we named the RFM pediatric (RFMp), may be useful as a simple tool to estimate whole-body fat percentage and as an alternative to BMI-for-age percentiles to assess body adiposity among girls and boys who are between 8 and 14 years of age. The RFM equation for adults may be useful as a tool to estimate whole-body fat percentage and as alternative to BMI-for-age percentiles to assess body adiposity among adolescents 15 to 19 years of age.

The simple RFMp linear equation was more accurate than the more complex BMI quadratic equation to estimate whole-body fat percentage among girls (88.2% vs. 85.7%; P = 0.027) and boys 8 to 14 years of age (83.4% vs. 71.0%; P < 0.001), across ethnic groups (Supplementary Table 4), and it had similar or superior accuracy to BMI, across body fat ranges (Supplementary Fig. 7B,E) and across age (Supplementary Fig. 8A,C). Among adolescents 15 to 19 years of age, RFM was more accurate than BMI among boys (82.3% vs. 73.9%; P < 0.001), including across ethnic groups. However, RFM was slightly less accurate than BMI among girls (89.0% vs. 92.6%; P = 0.002), including European-American girls and African-American girls but not Mexican-American girls (Supplementary Table 5). Both RFMp and RFM equations were more precise than BMI quadratic equations among boys.

TMI was developed as a powerful alternative tool to BMI to estimate body fat percentage and to better classify girls and boys with overweight compared with BMI-for-age percentiles^16^. Among children and adolescents 8 to 14 years of age, RFMp was more accurate than TMI to estimate whole-body fat percentage among boys but not girls. Among adolescents 15 to 19 years of age, RFM was more accurate than TMI among boys of Mexican, European and African ethnicity. TMI was more accurate than RFM among European-American girls but less accurate among African-American girls. Both RFM and TMI had equally high accuracy among Mexican-American girls. TMI are quadratic equations based on body weight and height. In contrast, RFMp and RFM are simple linear equations that show comparable accuracy to TMI equation to estimate whole-body fat percentage among girls. However, an advantage of RFMp and RFM is that the former equations are consistently more accurate than TMI to estimate whole-body fat percentage among boys. Thus, data from our study conducted in more than 10,000 children and adolescents and those from our previous study conducted in nearly 16,000 adult individuals^17^ strongly support the benefit of using height and waist circumference (the only anthropometrics required for RFMp and RFM calculations) over height and body weight for the purpose of assessing body fatness in children, adolescents and adult individuals. It should be noted that RFMp differs from the original RFM equation for adult individuals in their coefficients only^17^.

Our findings are consistent with a recent study showing waist-to-height Z scores were superior to BMI Z-scores to predict body fat percentage in children and adolescents^25^. WHtR has also been shown to be superior to BMI to predict whole-body fat percentage in adults^37^. However, in none of these previous studies misclassification error rate of overweight or obesity status was specifically evaluated.

It is known that WHtR is a better predictor of body fat in adult males than adult females^32,37^, and better in boys than girls^38^, which are also consistent with our findings in children and adolescents and those from our previous study in adult individuals^17^. WHtR is also a very useful predictor of cardiovascular risk factors in adults^39,40^ and children^41^. However, its use as an estimator of body fat percentage has been less studied in adults^37,42^ and children^26^. In fact, most of the studies in children have rather been focused on body fat prediction^18–21,23^ in relatively small populations. In contrast, our study was conducted in a representative sample of the U.S. pediatric population aged 8 to 19 years. Our findings show that RFMp and RFM equations are superior to WHtR in predicting whole-body fat percentage among African-American girls. It should be pointed out that one of the advantages of RFMp and RFM is that these equations provide a direct estimate of whole-body fat percentage, whereas the widely used BMI and WHtR indices do not.

In the population studied, our findings showed better diagnostic accuracy of overweight or obesity with RFMp than with BMI-for-age percentiles among both girls and boys 8 to 14 years of age. Among adolescents 15 to 19 years of age, RFM showed better diagnostic accuracy of overweight or obesity than BMI-for-age percentiles among boys but not girls. Among children and younger adolescents, total misclassification error rate of overweight or obesity (DXA-measured whole-body fat percentage ≥ 85^th^ percentile) was significantly lower for RFMp than for BMI-for-age percentiles among boys but not girls (Fig. 3). In addition, total misclassification error rate of overweight or obesity was significantly lower for RFMp than for BMI-for-age percentiles among African-American boys and girls. TMI, in contrast, showed no improvement over BMI-for-age percentiles in the rates of total misclassification error of overweight or obesity. We also found no improvement in misclassification error of overweight or obesity by TMI among non-Hispanic European-American boys and girls, which confirms the findings from a previous study in this ethnic group^16^.

Among adolescents 15 to 19 years of age, misclassification error rate of overweight or obesity was similar for RFM and BMI-for-age among girls and boys (Fig. 4). Conversely, TIM showed higher misclassification error than BMI-for-age percentiles among girls (Fig. 4A) but not boys (Fig. 4B). Both RFM and TMI had higher misclassification error than BMI-for-age percentiles among Mexican-American girls, suggesting that different diagnostic thresholds specific for Mexican-American girls 15 to 19 years of age and/or a modified equation specific for this subpopulation may be required, which warrants further investigation.

Although RFMp, RFM and BMI-for-age percentiles had similar rates of misclassification of overweight or obesity among girls, RFMp and RFM are simple linear equations which do not require adjustment for age as in the case of the complex BMI percentiles, which should be adjusted for age in months, according to the CDC recommendations^1,2^. More importantly, among boys 8 to 14 years of age, RFMp represented a significant improvement in misclassification error rate of overweight or obesity (1.4% lower) compared with BMI-for-age percentiles. Among African-American girls 8 to 14 years of age, RFMp also represented a significant improvement in overweight or obesity misclassification (3.1%) compared with BMI-for-age percentiles. Among adolescents 15 to 19 years of age, the total misclassification error of overweight or obesity was lower for RFM than for BMI-for-age percentiles among African-American boys (1.7% lower). Since our findings are derived from a representative sample of the U.S. population (24,897,743 children and adolescents aged 8 to 14 years of age and 17’932,184 adolescents aged 15 to 19 years of age at the time of the survey), this modest improvement with RFMp and RFM in fact would represent nearly 200,000 additional boys 8 to 14 years of age, 50,000 additional African-American girls, and 25,000 additional African-American boys 15 to 19 years of age who could benefit of earlier counselling and lifestyle intervention if correctly classified as having overweight or obesity, or could avoid stigmatization if correctly classified as not having overweight or obesity. Excess body fat in the early years of life predisposes to obesity in the adolescence^43^. Thus, the importance of an adequate assessment of body fatness in children is unquestionable. An undesirable consequence of stigmatizing children with obesity is that it may lead to negative changes in their behaviors such as binge eating, social isolation or avoidance of medical care^44^.

Our study has limitations: (1) We used DXA as the reference method for body fat percentage. DXA underestimates and overestimates body fat percentage among children and adolescents with lower and higher body fat percentage, respectively, compared against the most accurate non-invasive method available, the four-compartment method^45^. However, the linear relationship between both methods has been shown to be very high (R^2^ = 0.85) among children and adolescents^45^. (2) Our analysis by ethnicity was limited to Mexican-American, European-American, and African-American girls and boys between 8 and 19 years of age as DXA was performed only in participants who were 8 years of age and older, and NHANES did not oversample some minority ethnic groups. Thus, the generalizability of our findings should be limited to the ethnic groups studied and in the age between 8 and 19 years. (3) Our findings require external validation in large populations from other regions. (4) Accurate estimates of RFMp and RFM depend on reliable measurements of waist circumference. Although the intra-observer variability between anthropometric measurements is very high, inter-observer variability could be a problem^46^. Measurement error on waist circumference (and height) can be effectively reduced with proper training of healthcare providers^46,47^. (5) To date, waist circumference is still measured less often than body weight, which may limit the use of RFMp and RFM over BMI or the more recently suggested TMI. (6) Information on sexual maturation was not available for study participants. Age and sexual maturation are associated with changes in body composition in children and adolescents^48^. Thus, future studies are required to evaluate the accuracy performance of RFMp and RFM to estimate whole-body fat percentage across Tanner stages (sexual maturity rates). (7) Finally, prospective studies are required to create RFMp and RFM reference curves for children and adolescents based on cutoffs associated with cardiometabolic diseases linked to obesity. Since RFMp and RFM are based on the height/waist ratio, and leaner children will tend to have a smaller waist circumference, estimated whole-body fat percentage by these equations will tend to be zero or become negative if height/waist ratio value is unusually high. In our large dataset, we observed three negative values when using either RFMp or RFM. These negative values where identified in individuals who had a height/waist ratio ≥3.5 but were not excluded from analyses. However, observations with height/waist values > 3.5 represented only 0.029% of all children and adolescents studied. Thus, to minimize unreliable estimates of body fat percentage, we do not recommend using RFMp or RFM equations when the height/waist ratio is ≥3.

In conclusion, RFMp for children and adolescents 8 to 14 years of age and RFM for adolescents 15 to 19 years of age were useful simple linear equations to estimate whole-body fat percentage and diagnose body fat-defined overweight or obesity.

## Methods

### Study population

**Figure 5:**
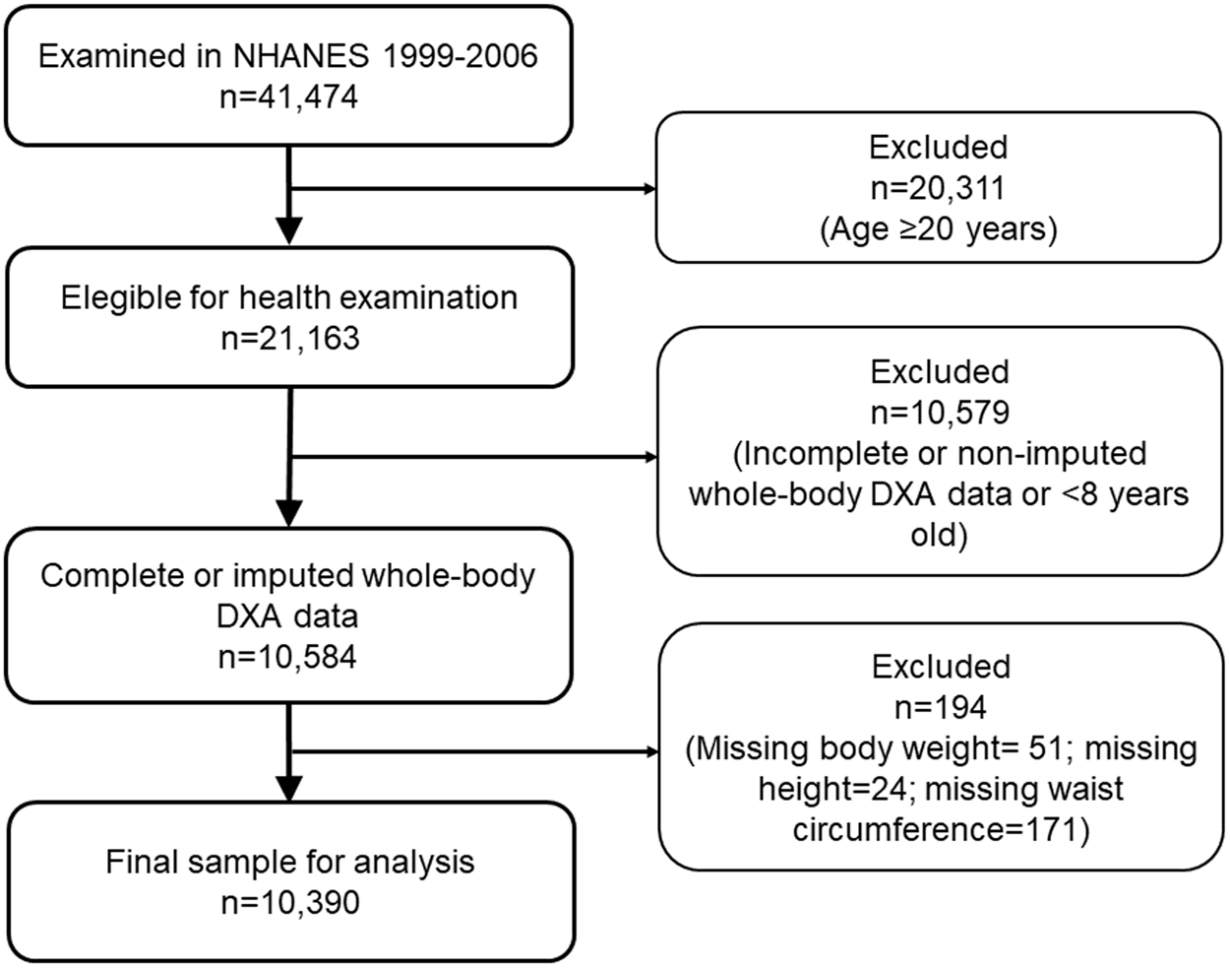
Flow diagram of participant selection. DXA, dual energy X-ray absorptiometry.

We studied American girls and boys 8 to 19 years of age who participated in the National Health and Nutrition Examination Survey (NHANES) from 1999 through 2006. Observations with missing data on body weight, height or waist circumference were excluded from the original dataset. The flow diagram of participant selection is shown in Fig. 5. NHANES uses a nationally representative sample selected using a multistage, probability sampling design^49^. The present study did not require approval or exemption from the Institutional Review Board at Cedars-Sinai Medical Center as it involved the use of publicly available de-identified data (https://wwwn.cdc.gov/nchs/nhanes/).

### Anthropometry and body composition

Anthropometric measurements were performed using standard procedures^50^. BMI was calculated as the body weight in kilograms divided by the square of the height in meters. Waist circumference was measured at the level of the uppermost lateral border of the right ilium during standing position. Body composition was measured by DXA using a Hologic QDR 4500A densitometer (Hologic, Inc., Bedford, Massachusetts). Whole-body fat percentage was calculated as the ratio between DXA-measured whole-body fat mass (g) and DXA-measured whole-body total mass (g), multiplied by 100. Multiple imputation was applied to replace missing DXA data. Five multiply imputed data were generated for each participant who had missing values^51^.

### Derivation of equations for pediatric populations

We used linear regression to derive equations for RFM and WHtR, and quadratic equations for BMI and TMI. The coefficients for RFM equations were rounded for practical purposes. Comparison was performed with WHtR, BMI and TMI raw (non-rounded) equations. Major differences in body composition were observed during the transition from childhood to adolescence and a relative stabilization of body fat percentage was noted in boys at the age of 15. Thus, we tested equations for different age categories. We performed sensitivity analysis to determine whether age categorization was ideal to achieve the highest performance of RFM equations.

### Whole-body fat prediction, estimation and agreement

Linear regression model was used to assess the prediction of whole-body fat percentage. Bias was calculated as the median difference between estimated and measured whole-body fat percentage. Accuracy was calculated as the percentage of cases with less than 20% difference between estimated and measured body fat percentage^52,53^. Precision was calculated as the interquartile range of the difference between estimated and measured body fat percentage^52–54^. These are accepted approaches used in clinical research to evaluate accuracy and precision^52–54^. Agreement between estimated and DXA-measured body fat percentage was evaluated using the concordance correlation coefficient (rho_c)^55^ and Bland-Altman plots^56^.

### Overweight and obesity misclassification

In clinical practice, overweight among school-aged children and adolescents is defined as a BMI ≥ 85^th^ percentile and <95^th^ percentile, which are specific for sex and age in months, as recommended by the Centers for Disease Control and Prevention (CDC)^2,57^. Obesity is defined as a BMI ≥ 95^th^ percentile^2,57^. Thus, comparison of misclassification error among indices was performed using their corresponding 85^th^ and 95^th^ percentiles calculated from the NHANES 1999-2006. Age in months at the time of physical examination was used for misclassification analysis to minimize the error in calculations. The diagnosis of overweight based on body fat percentage was arbitrarily set and defined as a DXA-measured body fat percentage ≥85^th^ percentile and <95^th^ percentile as suggested elsewhere^58^. The diagnosis of obesity based on body fat was also arbitrarily set and defined as a DXA-measured body fat percentage ≥95^th^ percentile^58^. Overweight and obesity diagnoses using TMI^16^, RFMp, RFM, and WHtR were also arbitrarily defined based on their corresponding 85^th^ and 95^th^ percentiles specific for sex (but not for age). We compared misclassification error rates of overweight, obesity and overweight or obesity for RFMp percentiles and RFM percentiles vs. BMI-for-age percentiles, TMI percentiles and WHtR percentiles. Misclassification was expressed as false negative rate (1−sensitivity), false positive rate (1−specificity), and total misclassification error rate, that is, the proportion of false positives and false negatives relative to the total population. Misclassification error rate of body fat-defined overweight, obesity and overweight or obesity for RFMp and RFM were compared with the misclassification error rates for BMI-for-age percentiles^2^, for TMI^16^, and WHtR.

Additionally, we compared misclassification error rates among BMI, TMI, RFMp and RFM using thresholds corresponding to the maximum Youden’s index, which maximizes the sum of sensitivity and specificity for a classifier, from the receiving operating characteristic curve^59^. Thresholds for BMI based on Youden’s index were adjusted for age.

### Diagnostic accuracy of overweight or obesity

As aforementioned, overweight or obesity was defined as a DXA-measured body fat percentage ≥85th percentile^58^. Diagnostic accuracy of overweight or obesity was estimated using the receiver-operating-characteristic curve analysis, expressed as the C-statistic.

### Association of RFMp and RFM with biomarkers for cardiometabolic disease

Correlation analysis (Pearson’s r) was performed between fasting plasma insulin, fasting plasma glucose, fasting serum LDL-cholesterol, fasting serum triacylglycerol (TAG), serum HDL-cholesterol (HDLc), serum total cholesterol, TAG/HDLc ratio and anthropometrics. Log-transformation was applied when appropriate. Listwise deletion was used to handle missing data.

### Statistical analysis

Information on clusters, strata and probability weights were used for all analyses to account for the complexity of the NHANES design, including response rate for physical examination (>80% for persons under the age of 20)^60^. Estimates of concordance correlation coefficients were adjusted for probability weights only. Pooled data estimates from multiple imputation were obtained using Rubin’s equations^61^, implemented for complex survey data^17^. Variance estimates for descriptive variables were obtained using Taylor series linearization. Bootstrapping with 1,000 replicates was used to obtain confidence intervals for adjusted R^2^ and the RMSE^17^. Presence of interactions were determined using the Wald test. All statistical analyses were performed using Stata 14 for Windows (StataCorp LP, College Station, TX). We set a P value less than 0.05 as statistically significant.

## Supplementary information

Supplementary Information

## References

[1] Ogden CL (2016). Trends in Obesity Prevalence Among Children and Adolescents in the United States, 1988-1994 Through 2013–2014. JAMA.

[2] Kuczmarski RJ (2002). 2000 CDC Growth Charts for the United States: methods and development. Vital Health Stat.

[3] Krebs NF (2007). Assessment of child and adolescent overweight and obesity. Pediatrics.

[4] Dulloo AG, Jacquet J, Solinas G, Montani JP, Schutz Y (2010). Body composition phenotypes in pathways to obesity and the metabolic syndrome. Int J Obes (Lond).

[5] Freedman DS (2005). The relation of childhood BMI to adult adiposity: the Bogalusa Heart Study. Pediatrics.

[6] Neovius MG, Linne YM, Barkeling BS, Rossner SO (2004). Sensitivity and specificity of classification systems for fatness in adolescents. Am J Clin Nutr.

[7] Sardinha LB, Going SB, Teixeira PJ, Lohman TG (1999). Receiver operating characteristic analysis of body mass index, triceps skinfold thickness, and arm girth for obesity screening in children and adolescents. Am J Clin Nutr.

[8] Cole TJ (1986). Weight/heightp compared to weight/height2 for assessing adiposity in childhood: influence of age and bone age on p during puberty. Ann Hum Biol.

[9] Heitmann BL, Erikson H, Ellsinger BM, Mikkelsen KL, Larsson B (2000). Mortality associated with body fat, fat-free mass and body mass index among 60-year-old swedish men-a 22-year follow-up. The study of men born in 1913. Int J Obes Relat Metab Disord.

[10] Ortega FB, Sui X, Lavie CJ, Blair SN (2016). Body Mass Index, the Most Widely Used But Also Widely Criticized Index: Would a Criterion Standard Measure of Total Body Fat Be a Better Predictor of Cardiovascular Disease Mortality?. Mayo Clin Proc.

[11] Padwal R, Leslie WD, Lix LM, Majumdar SR (2016). Relationship Among Body Fat Percentage, Body Mass Index, and All-Cause Mortality: A Cohort Study. Ann Intern Med.

[12] Must A, Phillips SM, Naumova EN (2012). Occurrence and timing of childhood overweight and mortality: findings from the Third Harvard Growth Study. J Pediatr.

[13] Franks PW (2010). Childhood obesity, other cardiovascular risk factors, and premature death. N Engl J Med.

[14] Reilly JJ, Kelly J (2011). Long-term impact of overweight and obesity in childhood and adolescence on morbidity and premature mortality in adulthood: systematic review. Int J Obes (Lond).

[15] Williams DP (1992). Body fatness and risk for elevated blood pressure, total cholesterol, and serum lipoprotein ratios in children and adolescents. Am J Public Health.

[16] Peterson CM (2017). Tri-Ponderal Mass Index vs Body Mass Index in Estimating Body Fat During Adolescence. JAMA Pediatr.

[17] Woolcott OO, Bergman RN (2018). Relative fat mass (RFM) as a new estimator of whole-body fat percentage ─ A cross-sectional study in American adult individuals. Sci Rep.

[18] Gutierrez Hervas AI, Cortes Castell E, Juste Ruiz M, Gil Guillen V, Rizo Baeza MM (2017). Estimation of body fat among 2-to-7-year-old Spanish children by different skinfolds equations and waist-to-height ratio. Nutr Hosp.

[19] Sijtsma A (2014). Waist-to-height ratio, waist circumference and BMI as indicators of percentage fat mass and cardiometabolic risk factors in children aged 3–7 years. Clin Nutr.

[20] Corvalan C, Uauy R, Kain J, Martorell R (2010). Obesity indicators and cardiometabolic status in 4-y-old children. Am J Clin Nutr.

[21] Santos S, Severo M, Lopes C, Oliveira A (2018). Anthropometric Indices Based on Waist Circumference as Measures of Adiposity in Children. Obesity (Silver Spring).

[22] Hubert H, Guinhouya CB, Allard L, Durocher A (2009). Comparison of the diagnostic quality of body mass index, waist circumference and waist-to-height ratio in screening skinfold-determined obesity among children. J Sci Med Sport.

[23] Frayon S (2018). Potential for waist-to-height ratio to detect overfat adolescents from a Pacific Island, even those within the normal BMI range. Obes Res Clin Pract.

[24] Sarria A (2001). Body mass index, triceps skinfold and waist circumference in screening for adiposity in male children and adolescents. Acta Paediatr.

[25] Tuan NT, Wang Y (2014). Adiposity assessments: agreement between dual-energy X-ray absorptiometry and anthropometric measures in U.S. children. Obesity (Silver Spring).

[26] Marrodan M (2014). Predicting percentage body fat through waist-to-height ratio (WtHR) in Spanish schoolchildren. Public Health Nutr.

[27] Jackson AS (2002). The effect of sex, age and race on estimating percentage body fat from body mass index: The Heritage Family Study. Int J Obes Relat Metab Disord.

[28] Newton RL (2006). Comparison of body composition methods in obese African-American women. Obesity (Silver Spring).

[29] Stevens J, Ou FS, Cai J, Heymsfield SB, Truesdale KP (2016). Prediction of percent body fat measurements in Americans 8 years and older. Int J Obes (Lond).

[30] Gomez-Ambrosi J (2012). Clinical usefulness of a new equation for estimating body fat. Diabetes Care.

[31] Lean ME, Han TS, Deurenberg P (1996). Predicting body composition by densitometry from simple anthropometric measurements. Am J Clin Nutr.

[32] Cui Z, Truesdale KP, Cai J, Stevens J (2014). Evaluation of anthropometric equations to assess body fat in adults: NHANES 1999–2004. Med Sci Sports Exerc.

[33] Friedl KE (2001). Evaluation of anthropometric equations to assess body-composition changes in young women. Am J Clin Nutr.

[34] Withers RT, Norton KI, Craig NP, Hartland MC, Venables W (1987). The relative body fat and anthropometric prediction of body density of South Australian females aged 17–35 years. Eur J Appl Physiol Occup Physiol.

[35] Gallagher D (2000). Healthy percentage body fat ranges: an approach for developing guidelines based on body mass index. Am J Clin Nutr.

[36] Lee DH (2017). Development and validation of anthropometric prediction equations for lean body mass, fat mass and percent fat in adults using the National Health and Nutrition Examination Survey (NHANES) 1999–2006. Br J Nutr.

[37] Swainson MG, Batterham AM, Tsakirides C, Rutherford ZH, Hind K (2017). Prediction of whole-body fat percentage and visceral adipose tissue mass from five anthropometric variables. PLoS One.

[38] Nambiar S, Hughes I, Davies PS (2010). Developing waist-to-height ratio cut-offs to define overweight and obesity in children and adolescents. Public Health Nutr.

[39] Ashwell M, Gunn P, Gibson S (2012). Waist-to-height ratio is a better screening tool than waist circumference and BMI for adult cardiometabolic risk factors: systematic review and meta-analysis. Obes Rev.

[40] Lee CM, Huxley RR, Wildman RP, Woodward M (2008). Indices of abdominal obesity are better discriminators of cardiovascular risk factors than BMI: a meta-analysis. J Clin Epidemiol.

[41] Lo K, Wong M, Khalechelvam P, Tam W (2016). Waist-to-height ratio, body mass index and waist circumference for screening paediatric cardio-metabolic risk factors: a meta-analysis. Obes Rev.

[42] Kagawa M, Byrne NM, Hills AP (2008). Comparison of body fat estimation using waist:height ratio using different ‘waist’ measurements in Australian adults. Br J Nutr.

[43] Geserick M (2018). Acceleration of BMI in Early Childhood and Risk of Sustained Obesity. N Engl J Med.

[44] Pont Stephen J., Puhl Rebecca, Cook Stephen R., Slusser Wendelin (2017). Stigma Experienced by Children and Adolescents With Obesity. Pediatrics.

[45] Sopher AB (2004). Measurement of percentage of body fat in 411 children and adolescents: a comparison of dual-energy X-ray absorptiometry with a four-compartment model. Pediatrics.

[46] Ulijaszek SJ, Kerr DA (1999). Anthropometric measurement error and the assessment of nutritional status. Br J Nutr.

[47] Panoulas VF (2008). The inter-operator variability in measuring waist circumference and its potential impact on the diagnosis of the metabolic syndrome. Postgrad Med J.

[48] Boot AM, Bouquet J, de Ridder MA, Krenning EP, de Muinck Keizer-Schrama SM (1997). Determinants of body composition measured by dual-energy X-ray absorptiometry in Dutch children and adolescents. Am J Clin Nutr.

[49] Curtin LR (2012). The National Health and Nutrition Examination Survey: Sample Design, 1999–2006. Vital Health Stat.

[50] National Health and Nutrition Examination Survey (NHANES). Anthropometry procedures manual. *Available from*, http://www.cdc.gov/nchs/data/nhanes/nhanes_07_08/manual_an.pdf, *Accessed May 16, 2016* (2007).

[51] National Health and Nutrition Examination Survey: Technical documentation for the 1999-2004. Dual Energy X-Ray Absorptiometry (DXA) multiple imputation data files. *Available from*, http://wwwn.cdc.gov/nchs/data/nhanes/dxa/dxa_techdoc.pdf, *Accessed May 17, 2016* (2008).

[52] Stevens LA, Zhang Y, Schmid CH (2008). Evaluating the performance of equations for estimating glomerular filtration rate. J Nephrol.

[53] Inker LA (2012). Estimating glomerular filtration rate from serum creatinine and cystatin C. N Engl J Med.

[54] Levey AS (2009). A new equation to estimate glomerular filtration rate. Ann Intern Med.

[55] Lin HM, Kim HY, Williamson JM, Lesser VM (2012). Estimating agreement coefficients from sample survey data. Survey Methodology.

[56] Bland JM, Altman DG (1999). Measuring agreement in method comparison studies. Stat Methods Med Res.

[57] CDC. Clinical Growth Charts. *Available at*, https://www.cdc.gov/growthcharts/clinical_charts.htm, *Accessed August 1*5*, 2018*. (2000).

[58] Ogden, C. L., Li, Y., Freedman, D. S., Borrud, L. G. & Flegal, K. M. Smoothed percentage body fat percentiles for U.S. children and adolescents, 1999–2004. *Natl Health Stat Report*, 1–7 (2011). 22164513

[59] Krzanowski, W. J. & Hand, D. J. *ROC curves for continuous data*. Vol. 111 (CRC Press, 2009).

[60] Johnson CL (2013). National health and nutrition examination survey: analytic guidelines, 1999–2010. Vital Health Stat.

[61] Rubin DB, Schenker N (1986). Multiple Imputation for Interval Estimation from Simple Random Samples with Ignorable Nonresponse. J Am Stat Assoc.

