# Supplementary material for: Relative Fat Mass as an estimator of whole-body fat percentage among children and adolescents: A cross-sectional study using NHANES
Source: Sci Rep. 2019 Oct 24;9:15279. doi: 10.1038/s41598-019-51701-z (PMC6813362; doi:10.1038/s41598-019-51701-z)
Supplement: Supplementary file 1 — Supplementary Information [file 41598_2019_51701_MOESM1_ESM.pdf]

Supplementary Material

Relative Fat Mass as an estimator of whole-body fat percentage among children and adolescents: A cross-sectional study using NHANES

Orison O. Woolcott, MD*, Richard N. Bergman, PhD

Sports Spectacular Diabetes and Obesity Wellness and Research Center, Cedars-Sinai Medical Center,

Los Angeles, CA 90048, USA

Running title: Estimation of body fat percentage

*Corresponding author:

Orison O. Woolcott, MD
Sports Spectacular Diabetes and Obesity Wellness and Research Center
Cedars-Sinai Medical Center
127 South San Vicente Blvd, AHSP A9220
Los Angeles, CA 90048
United States of America
Telephone: +1 (310) 423-2926
Email: Orison.Woolcott@cshs.org

Table of Contents

Supplementary Figure 1. Variability of anthropometrics and body composition by age among children and adolescents. 3

Supplementary Figure 2. RFMp and RFM values as functions of age. 4

Supplementary Figure 3. Prediction of whole-body fat percentage among girls by ethnicity. 5

Supplementary Figure 4. Prediction of whole-body fat percentage among boys by ethnicity. 6

Supplementary Figure 5. Agreement Bland-Altman plots between whole-body fat percentage estimated by RFMp (RFM pediatric) and that measured by DXA among children and adolescents 8 to 14 years of age. 7

Supplementary Figure 6. Agreement Bland-Altman plots between whole-body fat percentage estimated by RFM and that measured by DXA among adolescents 15 to 19 years of age. 8

Supplementary Figure 7. Performance of RFMp by quintiles of DXA-measured body fat percentage. 9

Supplementary Figure 8. Comparison of accuracy of RFMp and RFM linear equations with BMI and TMI quadratic equations. 10

Supplementary Figure 9. Performance of RFM by quintiles of DXA-measured body fat percentage. 11

Supplementary Figure 10. Comparison of total misclassification error rates in the diagnosis of overweight or obesity between indices among children and adolescents 8 to 14 years of age. 12

Supplementary Figure 11. Comparison of total misclassification error rates in the diagnosis of overweight or obesity between indices among adolescents 15 to 19 years of age. 13

Supplementary Table 1. Derived equations for the prediction of DXA-estimated whole-body fat percentage among participants (8 to 19 years of age) in NHANES 1999-2006. 14

Supplementary Table 2. Comparison of anthropometric indices for prediction of body fat percentage by sex and age among children and adolescents 8 to 14 years of age .* 16

Supplementary Table 3. Comparison of anthropometric indices for prediction of body fat percentage by sex and age among adolescents 15 to 19 years of age.* 17

Supplementary Table 4. Performance of RFMp by ethnicity to estimate body fat percentage in children and adolescents 8 to 14 years of age.* 18

Supplementary Table 5. Performance of RFM by ethnicity to estimate body fat percentage in adolescents 15 to 19 years of age.* 19

Supplementary Table 6. Youden's index cutoffs, false negative rate, false positive rate, and Youden's index for anthropometric indices to diagnose overweight and obesity among children and adolescents. 20

Supplementary Table 7. Correlation matrix (Pearson's r) between anthropometrics and biomarkers for cardiometabolic disease among children and adolescents 8 to 14 years of age. 21

Supplementary Table 8. Correlation matrix (Pearson's r) between anthropometrics and biomarkers for cardiometabolic disease among adolescents 15 to 19 years of age. 22

REFERENCE 23

Supplementary Figure 1. Variability of anthropometrics and body composition by age among children and adolescents.

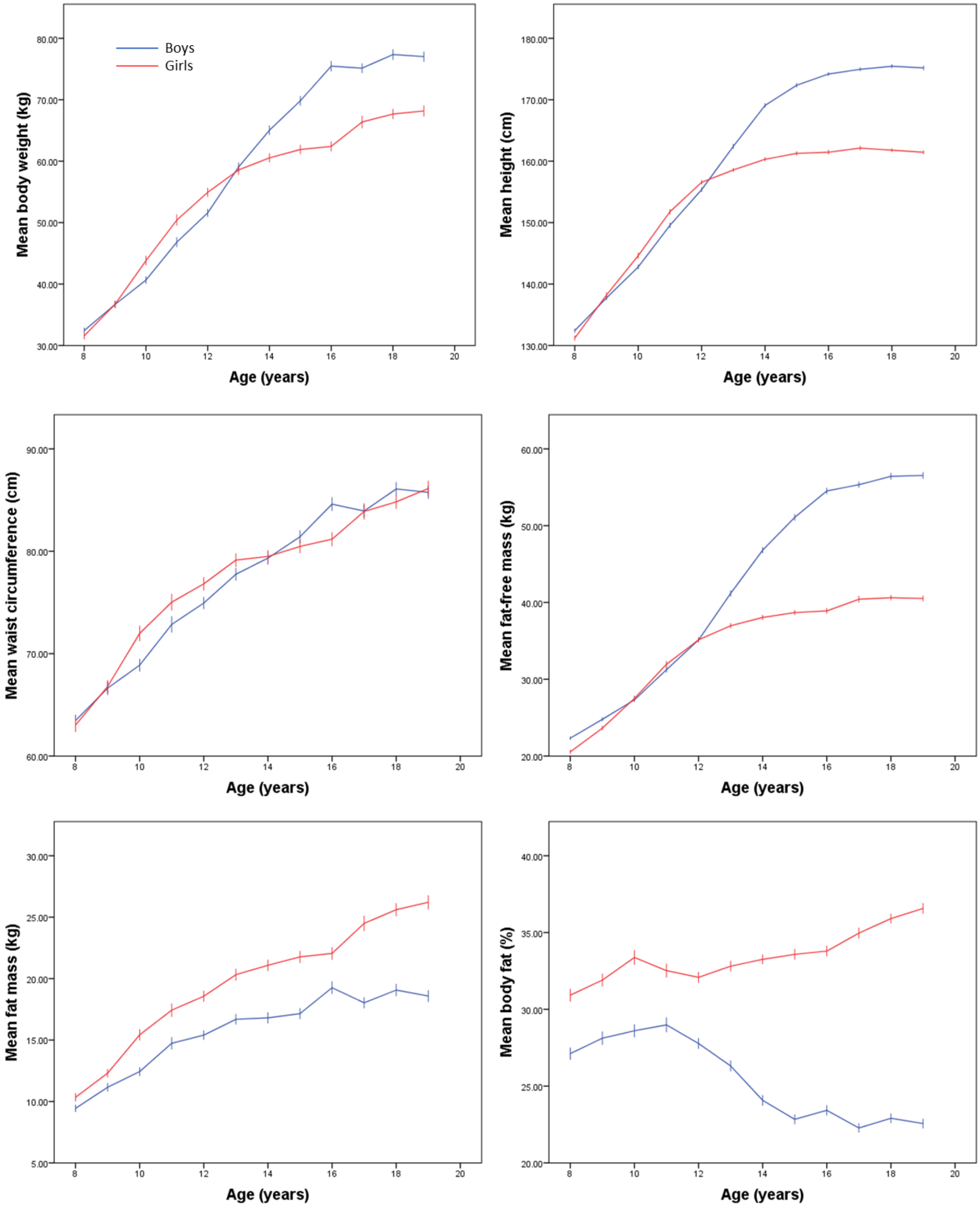

Supplementary Figure 2. RFMp and RFM values as functions of age.

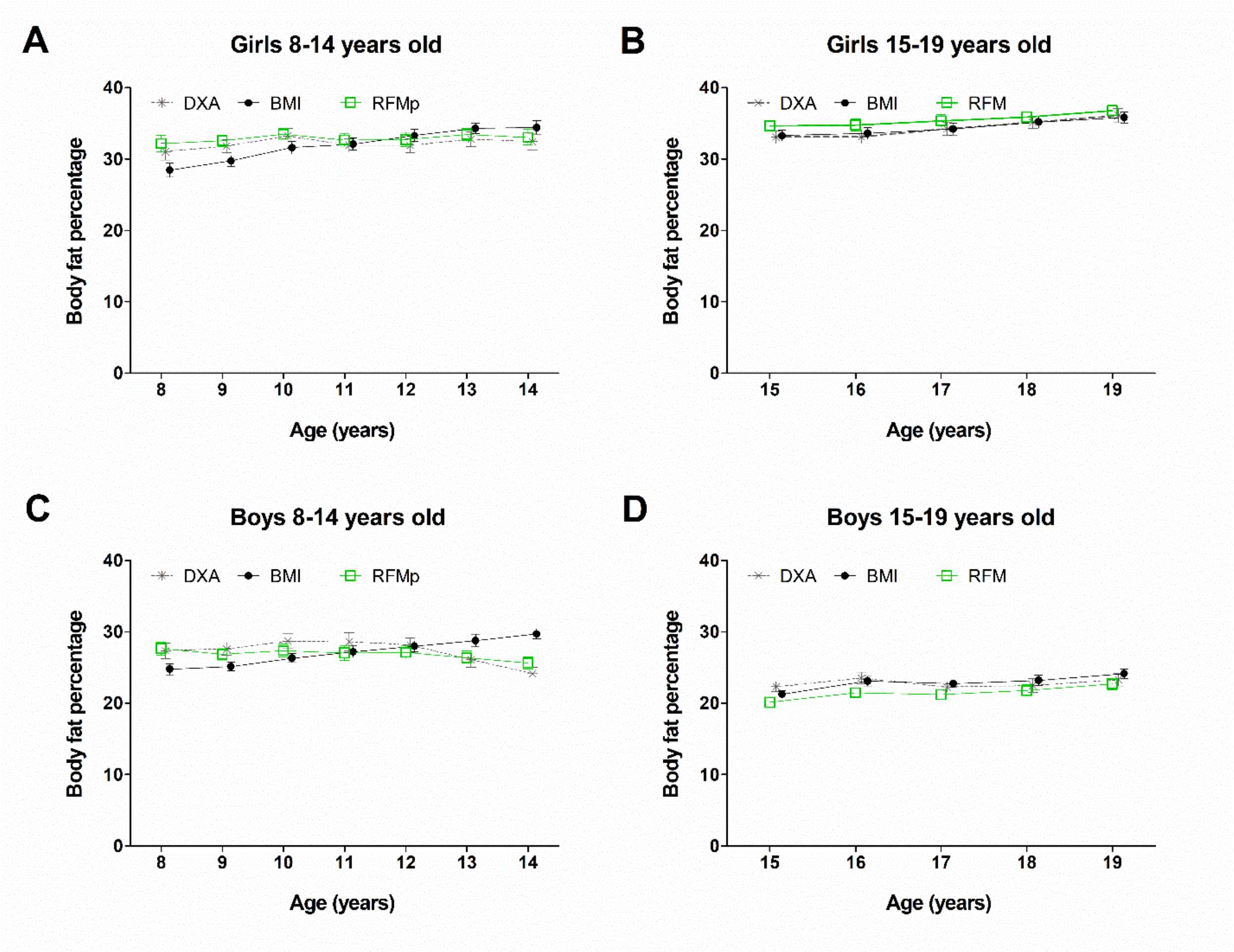

RFM, Relative Fat Mass; RFMp, Relative Fat Mass pediatric. Error bars are 95% confidence intervals.

Supplementary Figure 3. Prediction of whole-body fat percentage among girls by ethnicity.

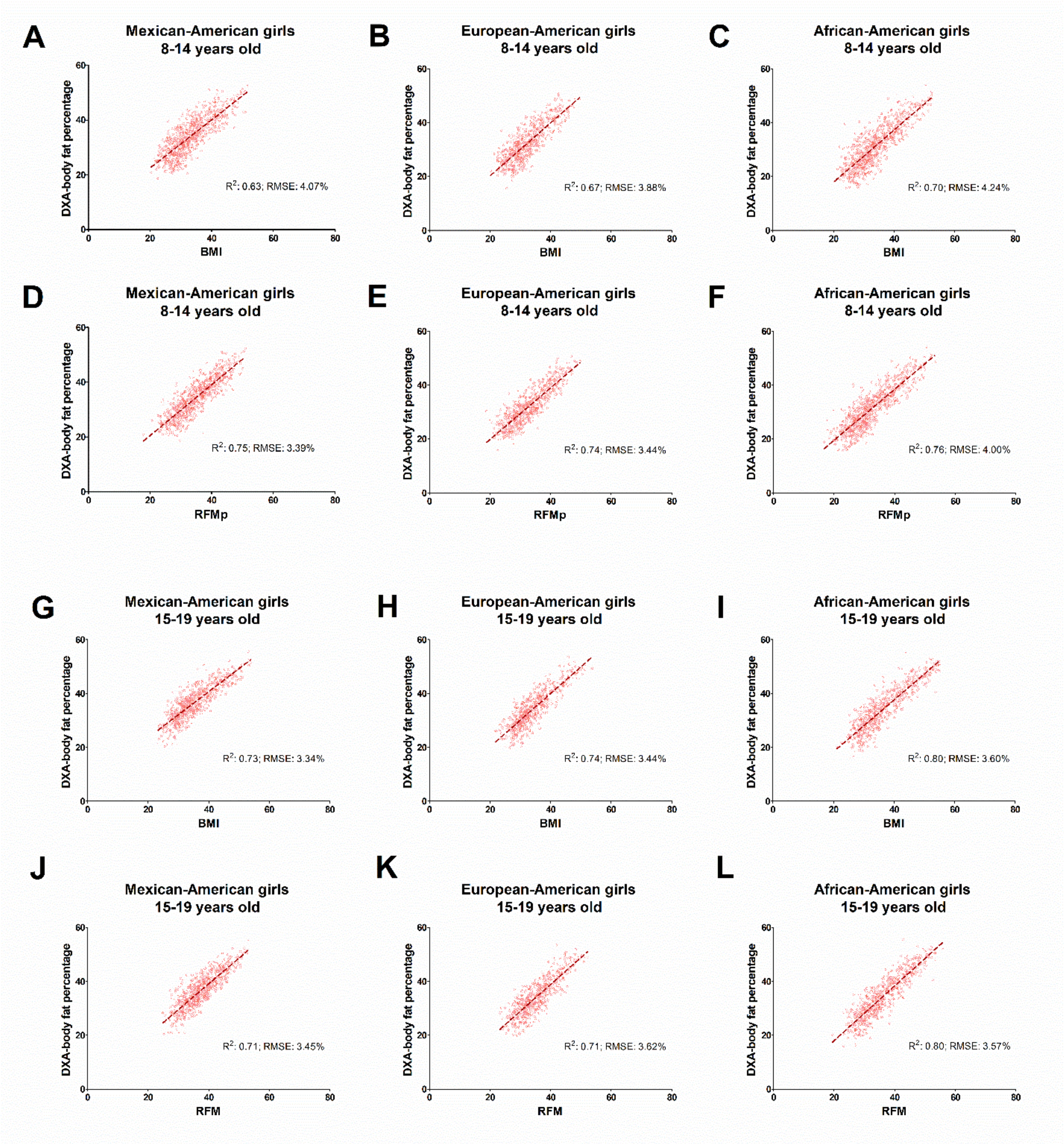

Supplementary Figure 4. Prediction of whole-body fat percentage among boys by ethnicity.

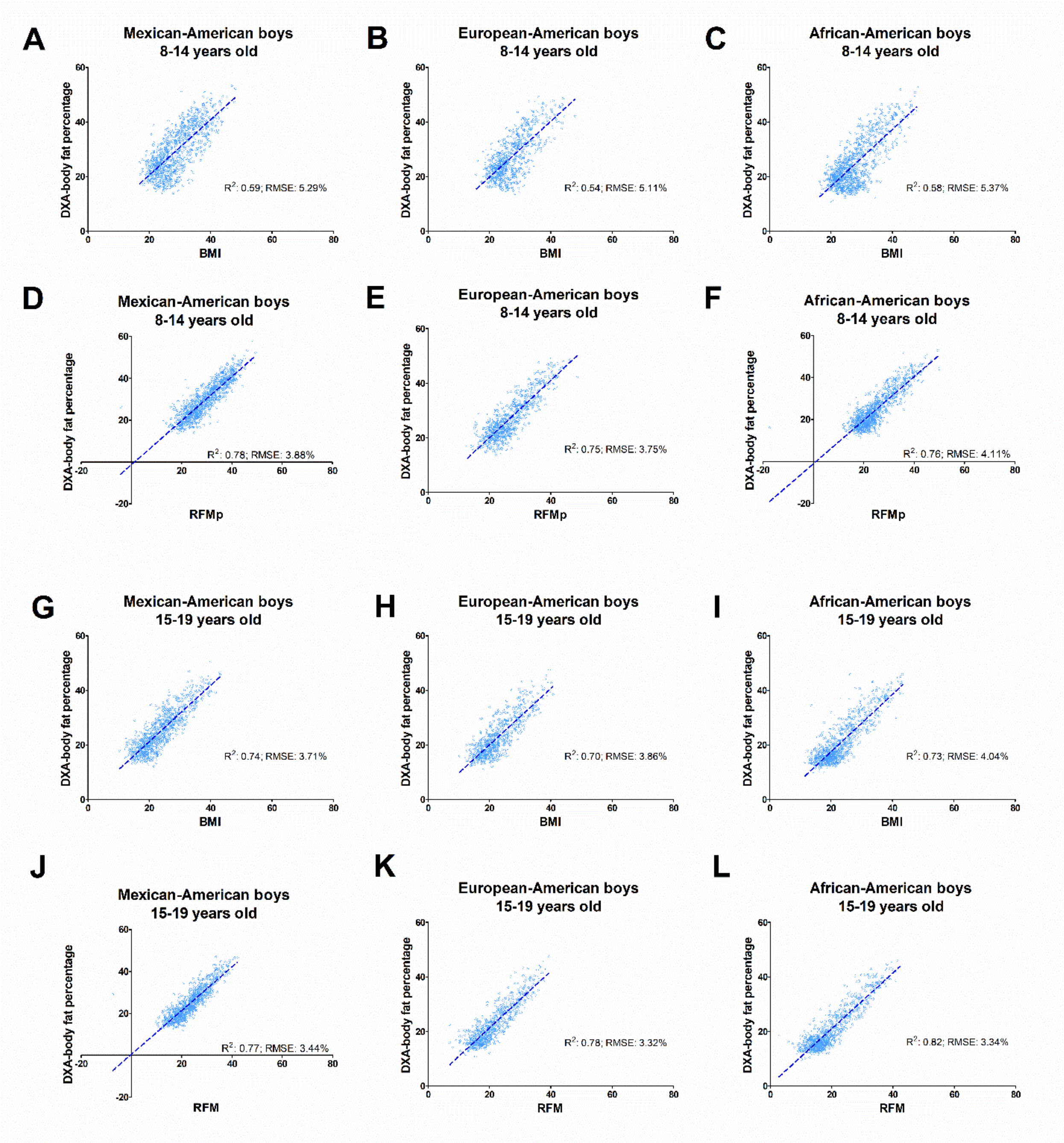

Supplementary Figure 5. Agreement Bland-Altman plots between whole-body fat percentage estimated by RFMp (RFM pediatric) and that measured by DXA among children and adolescents 8 to 14 years of age.

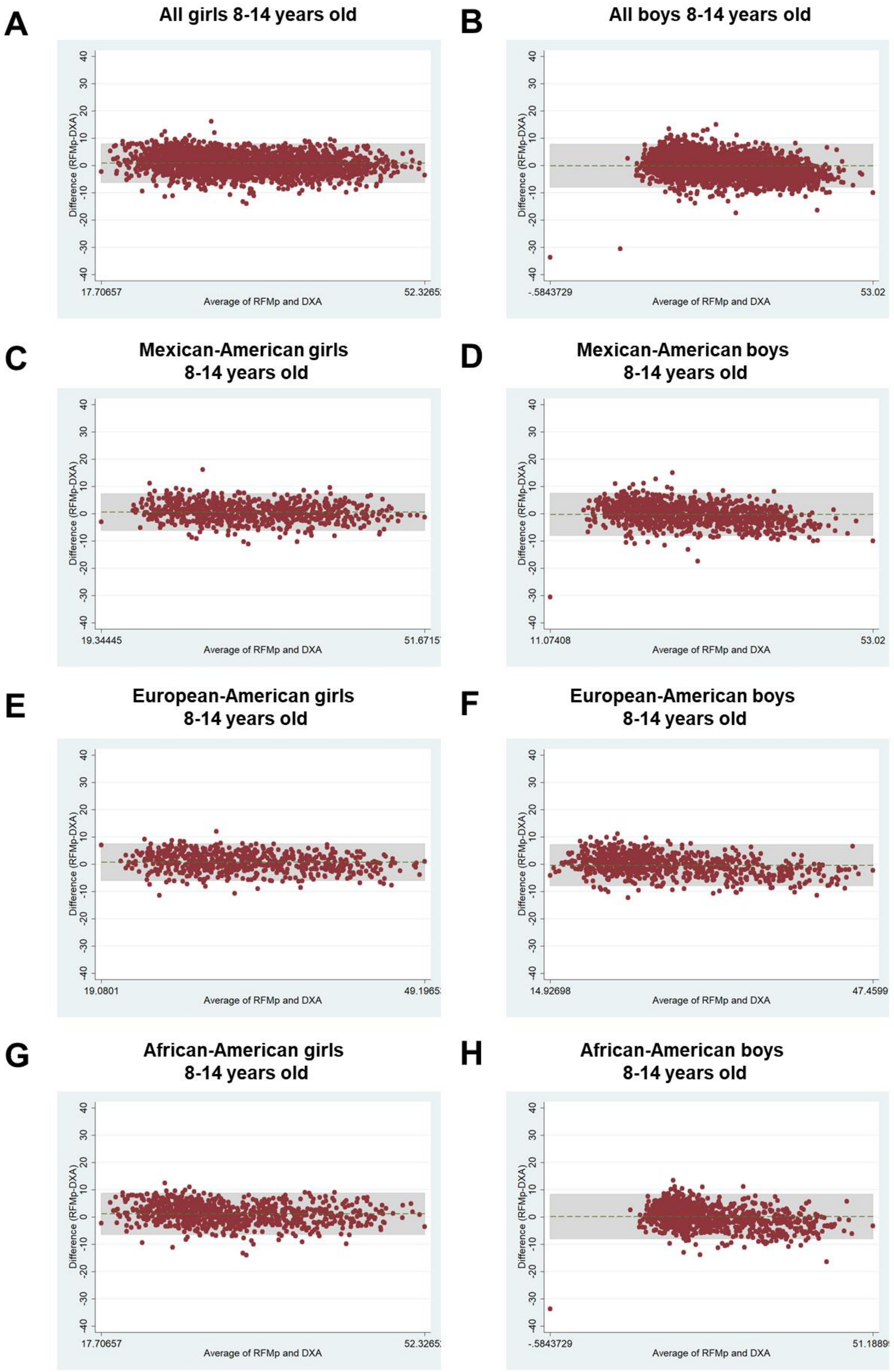

Gray bands indicate 95% limits of agreement. Green dotted lines represent the mean. Data plots correspond to DXA imputation 1.

Supplementary Figure 6. Agreement Bland-Altman plots between whole-body fat percentage estimated by RFM and that measured by DXA among adolescents 15 to 19 years of age.

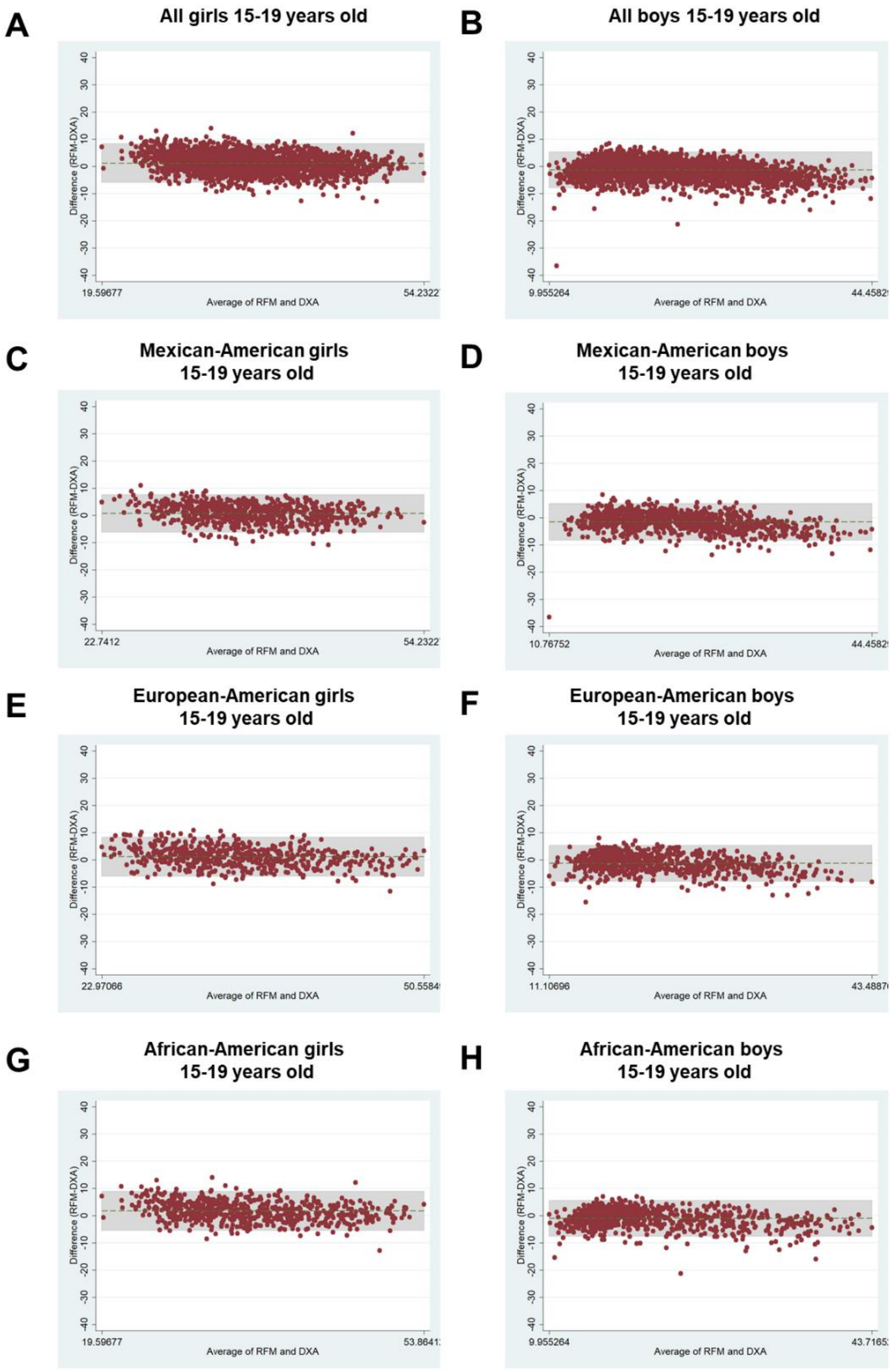

Gray bands indicate 95% limits of agreement. Green dotted lines represent the mean. Data plots correspond to DXA imputation 1.

Supplementary Figure 7. Performance of RFMp by quintiles of DXA-measured body fat percentage.

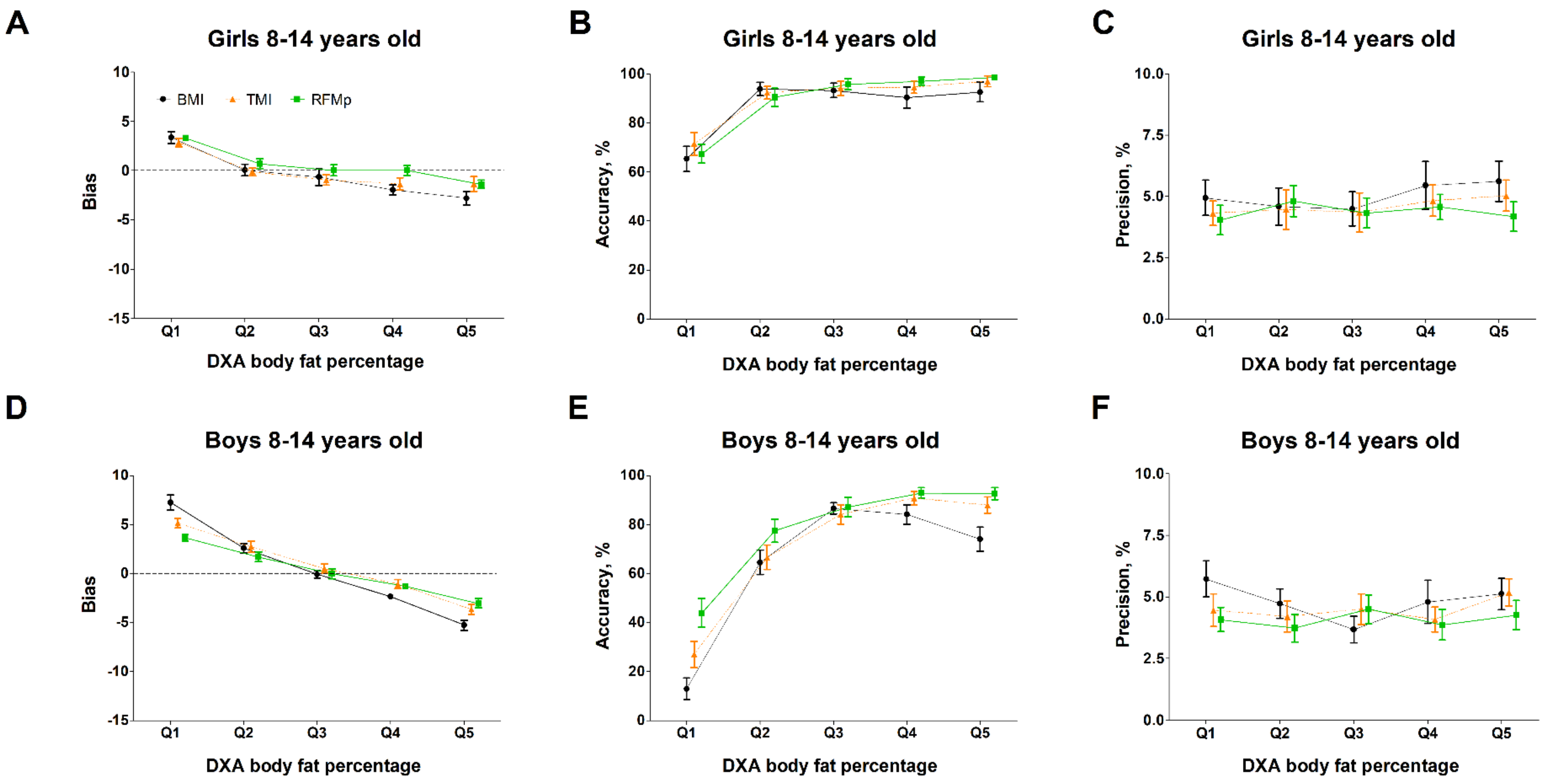

Bias was calculated as the median difference between estimated and measured body fat percentage. Accuracy was calculated as the percentage of cases with <20% difference between estimated and measured body fat percentage. Precision was calculated as the interquartile range of the difference between estimated and measured body fat percentage.
Error bars are 95% confidence intervals.

Supplementary Figure 8. Comparison of accuracy of RFMp and RFM linear equations with BMI and TMI quadratic equations.

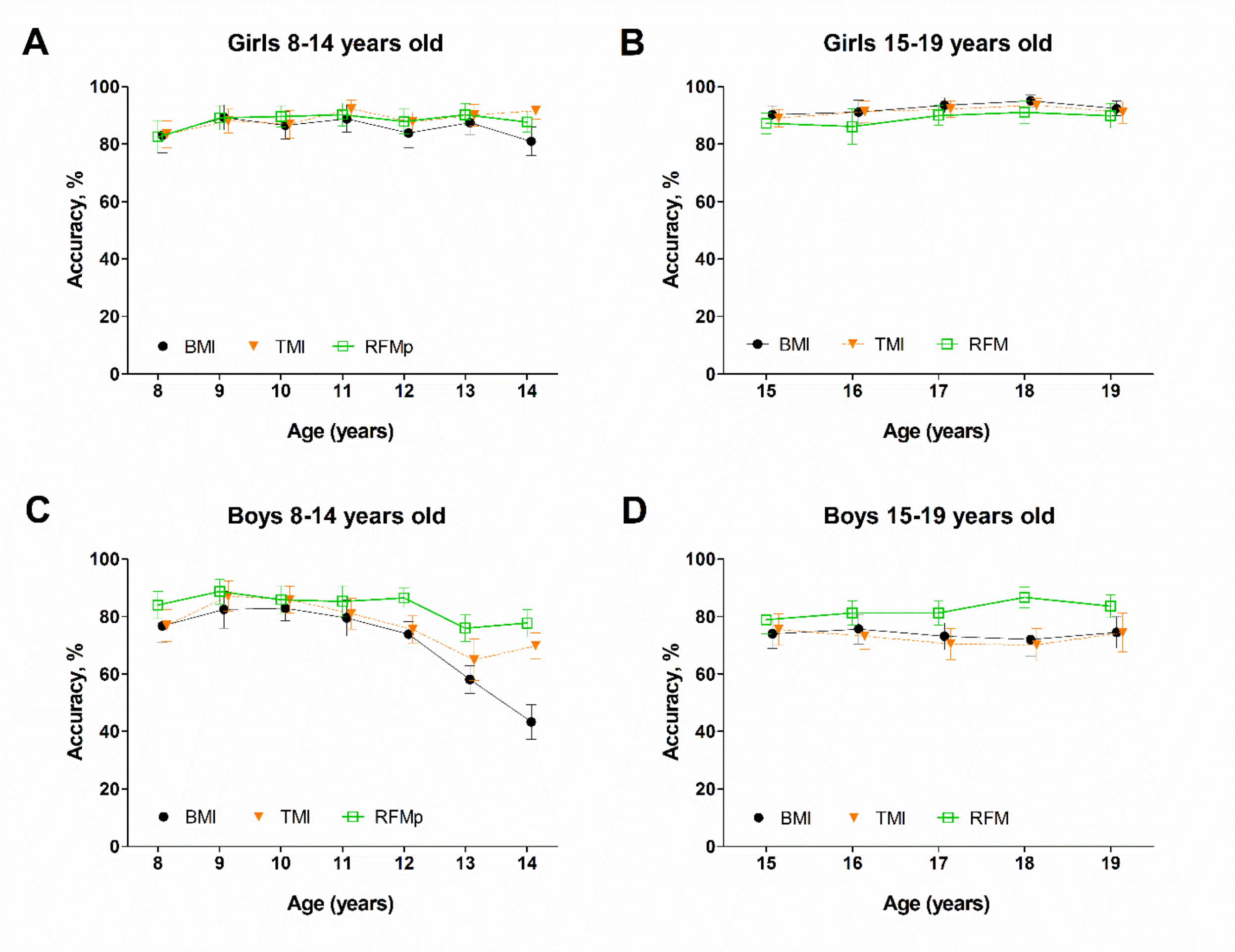

Error bars are 95% confidence intervals.

Supplementary Figure 9. Performance of RFM by quintiles of DXA-measured body fat percentage.

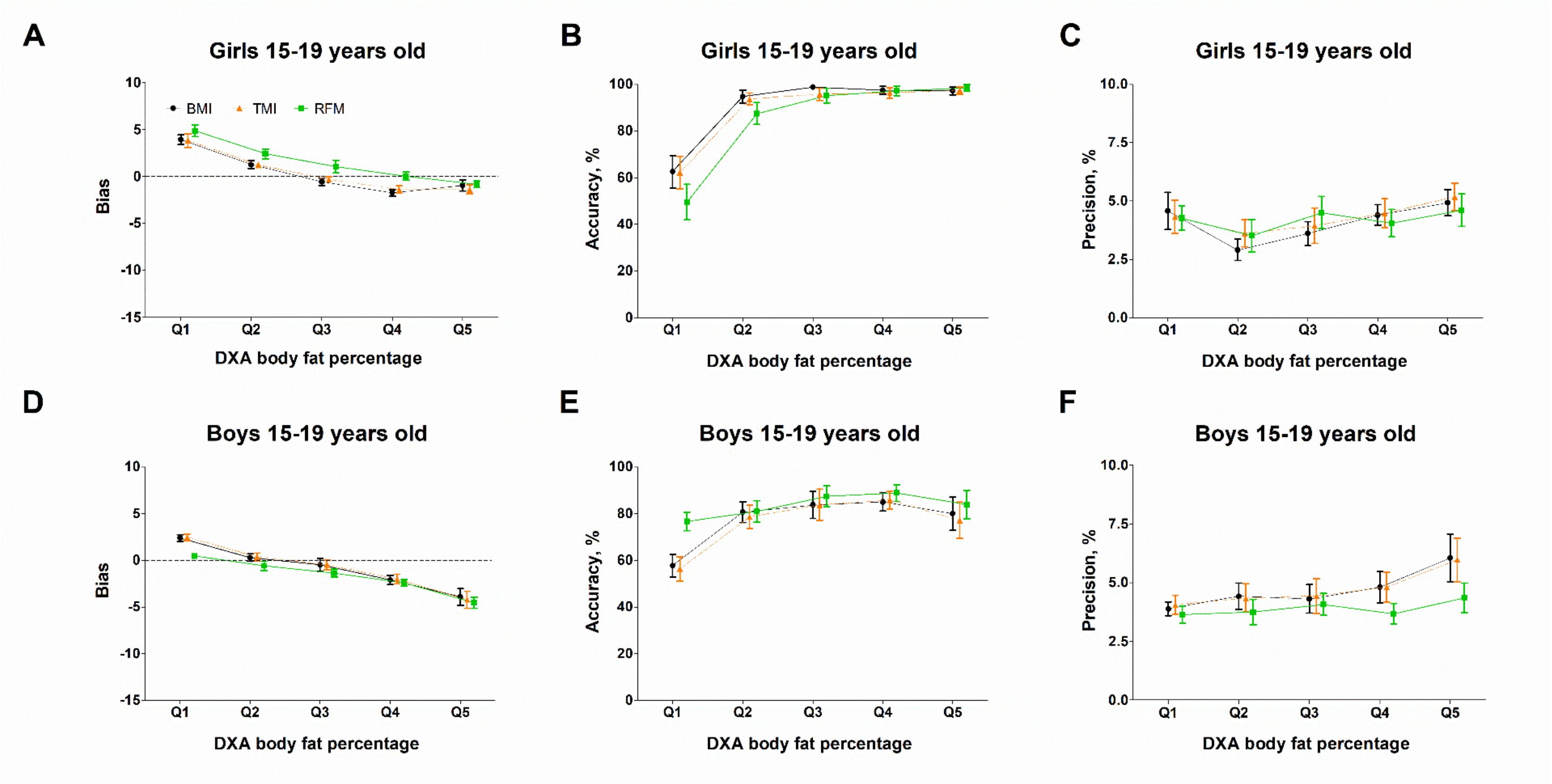

Bias was calculated as the median difference between estimated and measured body fat percentage. Accuracy was calculated as the percentage of cases with <20% difference between estimated and measured body fat percentage. Precision was calculated as the interquartile range of the difference between estimated and measured body fat percentage. Error bars are 95% confidence intervals.

Supplementary Figure 10. Comparison of total misclassification error rates in the diagnosis of overweight or obesity between indices among children and adolescents 8 to 14 years of age.

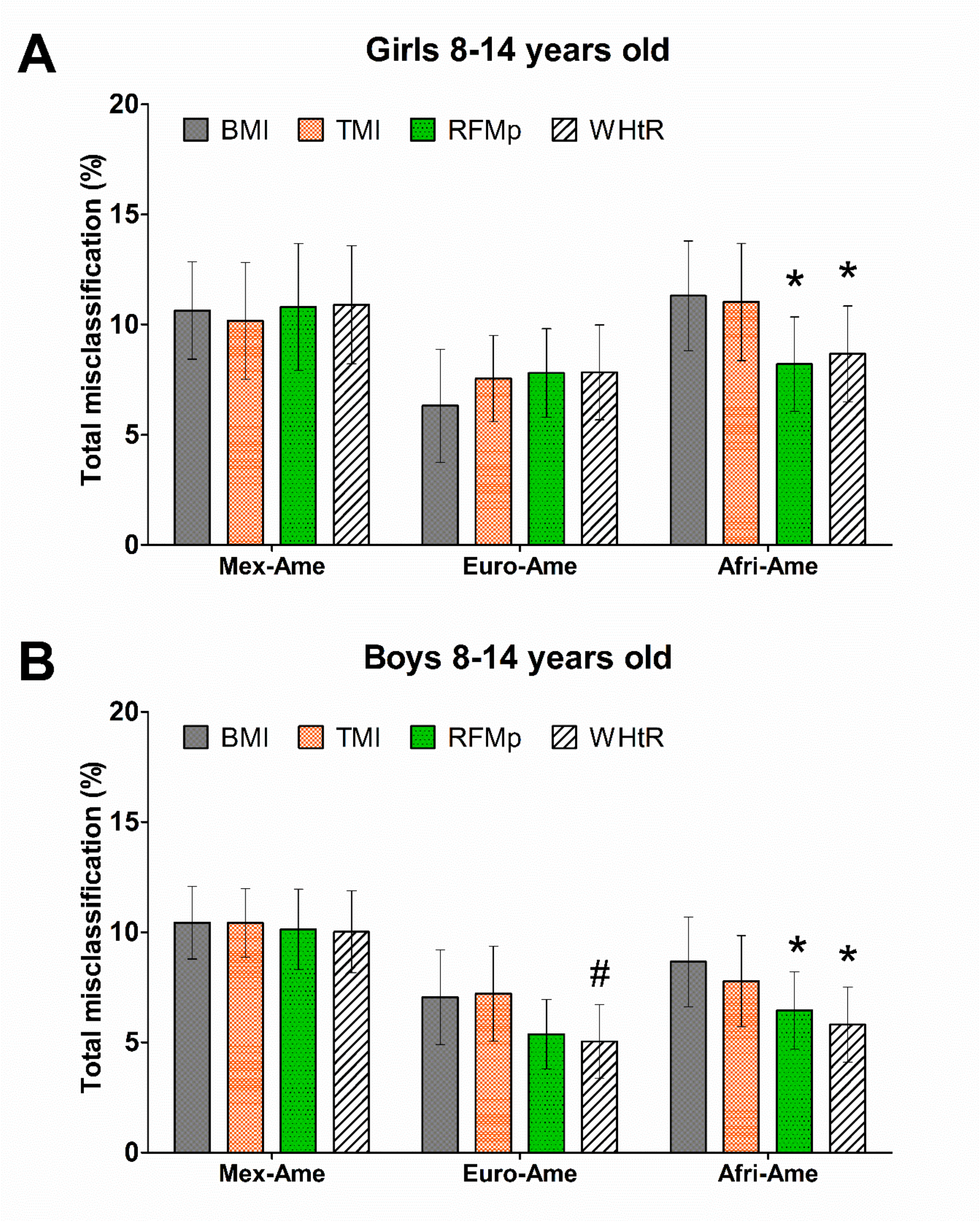

Error bars are 95% confidence intervals. Diagnostic cutoff values are 85th and 95th percentiles. *P<0.01; $^{\#}$P<0.05; compared with BMI.

Supplementary Figure 11. Comparison of total misclassification error rates in the diagnosis of overweight or obesity between indices among adolescents 15 to 19 years of age.

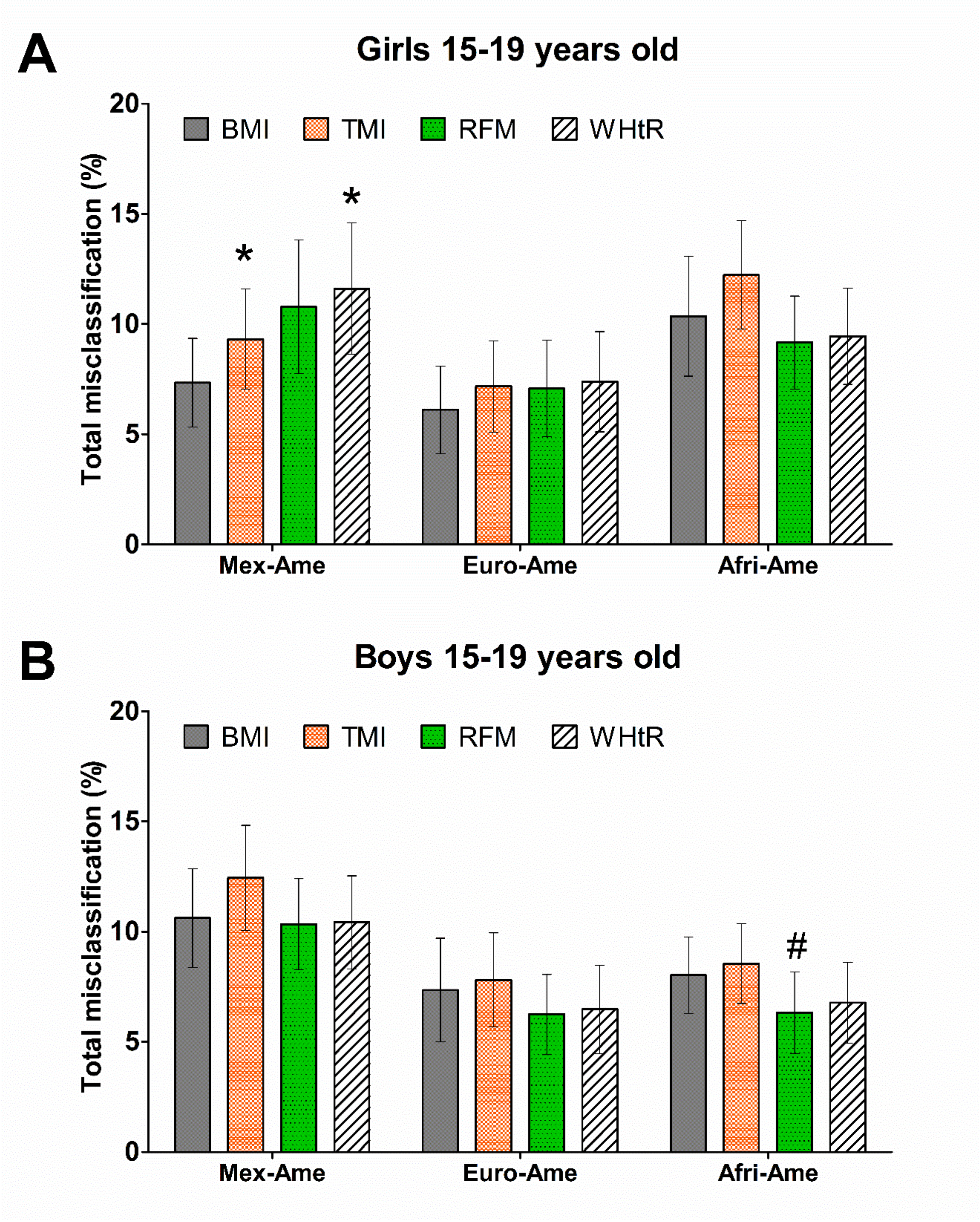

Error bars are 95% confidence intervals. Diagnostic cutoff values are 85th and 95th percentiles. *P<0.01; #P<0.05; compared with BMI.

Supplementary Table 1. Derived equations for the prediction of DXA-estimated whole-body fat percentage among participants (8 to 19 years of age) in NHANES 1999-2006.

**Equations for 8 to 14 years**	
**BMI**	
Equation for girls	–2.829 – (0.023 X $(\text{weight/height}^2)^2$) + (2.184 X (weight/height^2))
Equation for boys	–7.761 – (0.020 X $(\text{weight/height}^2)^2$) + (2.156 X (weight/height^2))
Equation for girls and boys	–7.338 – (0.021 X $(\text{weight/height}^2)^2$) + (2.161 X (weight/height^2)) + (4.16 X sex)
**TMI**	
Equation for girls	–12.376 – (0.075 X $(\text{weight/height}^3)^2$) + (4.305 X (weight/height^3))
Equation for boys	–20.062 – (0.071 X $(\text{weight/height}^3)^2$) + (4.516 X (weight/height^3))
Equation for girls and boys	–18.743 – (0.074 X $(\text{weight/height}^3)^2$) + (4.47 X (weight/height^3)) + (3.919 X sex)
**RFMp**	
Equation for girls	76.847 – (21.31 X (height/waist circumference))
Equation for boys	77.137 – (23.29 X (height/waist circumference))
Equation for girls and boys	75.446 – (22.5 X (height/waist circumference)) + (3.9 X sex)
**WHtR**	
Equation for girls	–8.291 + (83.154 X (waist circumference/height))
Equation for boys	–16.456 + (91.588 X (waist circumference/height))
Equation for girls and boys	–14.844 + (88.209 X (waist circumference/height)) + (4.093 X sex)
**Equations for 15 to 19 years of age**	
**BMI**	
Equation for girls	–7.082– (0.023 X $(\text{weight/height}^2)^2$) + (2.305 X (weight/height^2))
Equation for boys	–13.43 – (0.013 X $(\text{weight/height}^2)^2$) + (1.815 X (weight/height^2))
Equation for girls and boys	–16.284 – (0.018 X $(\text{weight/height}^2)^2$) + (2.073 X (weight/height^2)) + (11.734 X sex)
**TMI**	
Equation for girls	–5.636 – (0.058 X $(\text{weight/height}^3)^2$) + (3.601 X (weight/height^3))
Equation for boys	–12.747 – (0.037 X $(\text{weight/height}^3)^2$) + (3.107 X (weight/height^3))
Equation for girls and boys	–15.539– (0.054 X $(\text{weight/height}^3)^2$) + (3.561 X (weight/height^3)) + (9.62 X sex)
**RFM***	
Equation for adult women	73.071 – (18.554 X height/waist circumference)
Equation for adult men	64.980 – (20.469 X height/waist circumference)
Equation for adult women and men	62.979 – (19.361 X height/waist circumference) + (11.536 X sex)
**WHtR**	

Equation for girls	–0.342 + (68.629 X (waist circumference/height))
Equation for boys	–16.043 + (80.667 X (waist circumference/height))
Equation for girls and boys	–13.37 + (75.112 X (waist circumference/height)) + (9.747 X sex)

* Equations developed for adult individuals (≥20 years of age) [1].
TMI, tri-ponderal mass index; RFM, Relative Fat Mass; RFMp, Relative Fat Mass pediatric; BMI, body mass index (body weight in kilograms divided by squared height in meters). For TMI equations, body weight in kilograms, height in meters. WHtR, waist-to-height ratio.
For RFM equations, height and waist circumference are in same units.

Supplementary Table 2. Comparison of anthropometric indices for prediction of body fat percentage by sex and age among children and adolescents 8 to 14 years of age .*

	Age (years)						
	**8**	**9**	**10**	**11**	**12**	**13**	**14**
**Girls, n**	278	276	259	279	411	444	436
**R^2 (95% CI) †**							
BMI equation for girls 8 to 14 years of age	0.69	0.69	0.71	0.71	0.74	0.74	0.73
TMI equation for girls 8 to 14 years of age	0.67	0.69	0.75	0.72	0.71	0.72	0.72
RFM pediatric (RFMp) equation for girls 8 to 14 years of age	0.71	0.69	0.78	0.77	0.76	0.76	0.73
WHtR equation for girls 8 to 14 years of age	0.73	0.68	0.77	0.76	0.76	0.74	0.73
**RMSE (95% CI) ‡**							
BMI equation for girls 8 to 14 years of age	3.77	3.62	4.02	3.73	3.57	3.57	3.63
TMI equation for girls 8 to 14 years of age	3.91	3.66	3.78	3.66	3.78	3.65	3.72
RFM pediatric (RFMp) equation for girls 8 to 14 years of age	3.66	3.61	3.56	3.35	3.42	3.37	3.63
WHtR equation for girls 8 to 14 years of age	3.52	3.67	3.61	3.41	3.45	3.57	3.66
**Boys, n**	358	350	362	330	589	567	529
**R^2 (95% CI) †**							
BMI equation for boys 8 to 14 years of age	0.73	0.80	0.77	0.71	0.68	056	0.61
TMI equation for boys 8 to 14 years of age	0.68	0.80	0.76	0.70	0.72	0.62	0.67
RFM pediatric (RFMp) equation for boys 8 to 14 years of age	0.74	0.78	0.79	0.78	0.81	0.75	0.77
WHtR equation for boys 8 to 14 years of age	0.75	0.80	0.79	0.78	0.80	0.74	0.77
**RMSE (95% CI) ‡**							
BMI equation for boys 8 to 14 years of age	3.80	3.30	3.68	4.42	4.78	5.37	4.63
TMI equation for boys 8 to 14 years of age	4.13	3.44	3.74	4.54	4.44	5.00	4.30
RFM pediatric (RFMp) equation for boys 8 to 14 years of age	3.74	3.42	3.50	3.91	3.66	4.01	3.61
WHtR equation for boys 8 to 14 years of age	3.65	3.25	3.49	3.88	3.81	4.14	3.55

* Values represent pooled weighted estimates from DXA imputed data.
† R^2, coefficient of determination.
‡ RMSE, root mean squared error.

Supplementary Table 3. Comparison of anthropometric indices for prediction of body fat percentage by sex and age among adolescents 15 to 19 years of age.*

	Age (years)				
	**15**	**16**	**17**	**18**	**19**
**Girls, n**	391	371	389	514	445
**R^2 (95% CI) †**					
BMI equation for girls 15 to 19 years of age	0.73	0.68	0.70	0.76	0.73
TMI equation for girls 15 to 19 years of age	0.72	0.67	0.71	0.75	0.71
RFM equation for adult women	0.75	0.66	0.73	0.75	0.69
WHtR equation for girls 15 to 19 years of age	0.74	0.67	0.70	0.72	0.67
**RMSE (95% CI) ‡**					
BMI equation for girls 15 to 19 years of age	3.52	3.60	3.50	3.42	3.77
TMI equation for girls 15 to 19 years of age	3.58	3.63	3.42	3.48	3.93
RFM equation for adult women	3.36	3.66	3.31	3.53	4.02
WHtR equation for girls 15 to 19 years of age	3.47	3.63	3.47	3.68	4.20
**Boys, n**	550	609	588	544	521
**R^2 (95% CI) †**					
BMI equation for boys 15 to 19 years of age	0.69	0.73	0.66	0.72	0.74
TMI equation for boys 15 to 19 years of age	0.69	0.72	0.61	0.71	0.73
RFM equation for adult men	0.79	0.80	0.75	0.82	0.81
WHtR equation for boys 15 to 19 years of age	0.80	0.81	0.76	0.82	0.83
**RMSE (95% CI) ‡**					
BMI equation for boys 15 to 19 years of age	4.01	4.00	3.95	3.71	3.77
TMI equation for boys 15 to 19 years of age	3.99	4.08	4.23	3.78	3.85
RFM equation for adult men	3.27	3.48	3.38	3.01	3.18
WHtR equation for boys 15 to 19 years of age	3.20	3.41	3.33	2.98	2.99

* Values represent pooled weighted estimates from DXA imputed data.
† R^2, coefficient of determination.
‡ RMSE, root mean squared error.

Supplementary Table 4. Performance of RFMp by ethnicity to estimate body fat percentage in children and adolescents 8 to 14 years of age.*****

	**All**	**Mexican-American**	**European-American**	**African-American**
**Girls**				
**Bias (95% CI)†**				
BMI	−0.1 (−0.4 to 0.2)	−1.2 (−1.7 to −0.7)	−0.4 (−0.7 to −0.1)	2.4 (2.0 to 2.7)
TMI	0.0 (−0.3 to 0.3)	−0.2 (−0.7 to 0.2)	−0.3 (−0.6 to 0.0)	2.3 (2.0 to 2.7)
RFMp	0.7 (0.4 to 1.0)	0.7 (0.4 to 0.9)	0.8 (0.4 to 1.1)	1.2 (0.7 to 1.6)
**Accuracy, % (95% CI)‡**				
BMI	85.6 (83.7 to 87.6)	87.9 (85.4 to 90.4)	87.8 (84.9 to 90.6)	77.1 (74.4 to 79.8)
TMI	88.7 (87.3 to 90.0)	91.3 (89.1 to 93.5)	91.1 (88.8 to 93.4)	77.5 (74.4 to 80.7)
RFMp	88.2 (86.5 to 89.9)	91.3 (88.8 to 93.8)	88.9 (86.1 to 91.7)	83.8 (81.3 to 86.3)
**Precision, % (95% CI)§**				
BMI	5.71 (5.31 to 6.12)	5.79 (5.28 to 6.31)	5.34 (4.76 to 5.92)	5.60 (5.06 to 6.14)
TMI	5.21 (4.87 to 5.55)	5.08 (4.66 to 5.49)	4.79 (4.31 to 5.26)	5.46 (4.98 to 5.94)
RFMp	5.00 (4.73 to 5.28)	4.52 (4.11 to 4.92)	4.81 (4.41 to 5.21)	5.21 (4.78 to 5.65)
**Boys**				
**Bias (95% CI)**				
BMI	−0.3 (−0.7 to 0.1)	−2.1 (−2.5 to −1.6)	−0.7 (−1.2 to −0.1)	2.3 (1.9 to 2.7)
TMI	0.0 (−0.3 to 0.3)	−0.5 (−0.8 to −0.2)	−0.4 (−0.7 to −0.1)	2.7 (2.2 to 3.2)
RFMp	−0.4 (−0.7 to −0.1)	−0.4 (−0.8 to −0.1)	−0.4 (−0.9 to 0.0)	0.1 (−0.3 to 0.5)
**Accuracy, % (95% CI)**				
BMI	71.0 (68.8 to 73.3)	73.3 (70.4 to 76.1)	73.4 (70.3 to 76.4)	60.4 (56.9 to 63.9)
TMI	77.3 (75.1 to 79.4)	80.7 (77.6 to 83.7)	80.1 (77.0 to 83.2)	61.9 (58.6 to 65.3)
RFMp	83.4 (81.5 to 85.4)	86.2 (83.9 to 88.6)	84.3 (81.5 to 87.2)	76.5 (73.4 to 79.7)
**Precision, % (95% CI)**				
BMI	7.16 (6.72 to 7.59)	7.49 (6.83 to 8.16)	6.67 (5.92 to 7.42)	7.20 (6.59 to 7.81)
TMI	5.98 (5.60 to 6.35)	5.76 (5.21 to 6.31)	5.75 (5.21 to 6.28)	5.75 (5.25 to 6.26)
RFMp	5.01 (4.67 to 5.35)	5.00 (4.64 to 5.37)	4.90 (4.44 to 5.36)	5.47 (5.04 to 5.90)

* Values represent weighted estimates with 95% confidence intervals (95% CI) from DXA imputed data.
† Bias was calculated as the median difference between estimated and measured body fat percentage.
‡ Accuracy was calculated as the percentage of cases with <20% difference between estimated and measured body fat percentage.
§ Precision was calculated as the interquartile range of the difference between estimated and measured body fat percentage.

Supplementary Table 5. Performance of RFM by ethnicity to estimate body fat percentage in adolescents 15 to 19 years of age.*

	All	Mexican-American	European-American	African-American
**Girls**				
**Bias (95% CI)†**				
BMI	0.0 (−0.3 to 0.3)	−1.2 (−1.6 to −0.8)	−0.1 (−0.5 to 0.3)	2.2 (1.8 to 2.5)
TMI	0.0 (−0.2 to 0.3)	−0.5 (−0.9 to −0.1)	−0.4 (−0.8 to 0.0)	2.0 (1.7 to 2.4)
RFM	1.1 (0.9 to 1.4)	1.1 (0.8 to 1.5)	1.1 (0.7 to 1.4)	1.6 (1.2 to 2.1)
**Accuracy, % (95% CI)‡**				
BMI	92.6 (91.1 to 94.1)	94.6 (93.1 to 96.1)	93.7 (91.3 to 96.0)	85.4 (82.5 to 88.3)
TMI	91.5 (90.0 to 93.0)	93.8 (92.1 to 95.6)	92.6 (90.3 to 94.8)	84.1 (80.9 to 87.2)
RFM	89.0 (86.7 to 91.2)	92.9 (90.9 to 94.8)	89.2 (86.1 to 92.3)	86.7 (84.2 to 89.2)
**Precision, % (95% CI)§**				
BMI	4.78 (4.52 to 5.05)	4.51 (4.16 to 4.87)	4.58 (4.10 to 5.07)	4.62 (4.20 to 5.04)
TMI	4.76 (4.43 to 5.08)	4.80 (4.49 to 5.10)	4.68 (4.25 to 5.11)	4.93 (4.50 to 5.37)
RFM	4.64 (4.35 to 4.93)	4.80 (4.44 to 5.15)	4.57 (4.10 to 5.04)	4.81 (4.43 to 5.18)
**Boys**				
**Bias (95% CI)**				
BMI	0.4 (0.2 to 0.7)	−1.2 (−1.5 to −1.0)	0.4 (0.1 to 0.7)	3.0 (2.7 to 3.3)
TMI	0.3 (0.0 to 0.6)	−0.7 (−1.0 to −0.4)	0.1 (−0.4 to 0.6)	2.8 (2.5 to 3.1)
RFM	−1.1 (−1.4 to −0.8)	−1.5 (−1.8 to −1.3)	−1.1 (−1.5 to −0.7)	−0.6 (−0.9 to −0.4)
**Accuracy, % (95% CI)**				
BMI	73.9 (71.3 to 76.5)	79.2 (76.3 to 82.1)	76.7 (73.4 to 79.9)	53.0 (49.4 to 56.6)
TMI	72.8 (70.0 to 75.6)	80.5 (78.0 to 83.0)	74.0 (70.3 to 77.6)	54.8 (51.3 to 58.4)
RFM	82.3 (80.3 to 84.2)	86.1 (83.6 to 88.7)	83.3 (80.5 to 86.1)	79.5 (77.3 to 81.6)
**Precision, % (95% CI)**				
BMI	5.09 (4.79 to 5.39)	4.76 (4.29 to 5.22)	4.95 (4.46 to 5.44)	5.17 (4.85 to 5.48)
TMI	5.25 (4.94 to 5.56)	5.15 (4.69 to 5.61)	5.21 (4.81 to 5.60)	5.39 (4.99 to 5.80)
RFM	4.51 (4.21 to 4.80)	4.34 (4.02 to 4.65)	4.53 (4.09 to 4.97)	4.29 (3.92 to 4.67)

* Values represent weighted estimates with 95% confidence intervals (95% CI) from DXA imputed data.
† Bias was calculated as the median difference between estimated and measured body fat percentage.
‡ Accuracy was calculated as the percentage of cases with <20% difference between estimated and measured body fat percentage.
§ Precision was calculated as the interquartile range of the difference between estimated and measured body fat percentage.

Supplementary Table 6. Youden's index cutoffs, false negative rate, false positive rate, and Youden's index for anthropometric indices to diagnose overweight and obesity among children and adolescents.

**Overweight***				
	**Cutoff**	**FNR (%)**	**FPR (%)**	**Youden's index**
**Girls 8 to 14 years of age**				
BMI	21.5	9.5	29.1	0.61
TMI	15.5	14.0	17.2	0.69
RFMp	36.1	5.8	23.1	0.71
**Boys 8 to 14 years of age**				
BMI	22.3	11.0	17.7	0.71
TMI	14.5	6.8	19.7	0.73
RFMp	31.6	8.9	17.1	0.74
**Girls 15 to 19 years of age**				
BMI	26.2	8.3	19.6	0.72
TMI	16.0	7.7	21.6	0.71
RFM	38.7	7.7	19.8	0.73
**Boys 15 to 19 years of age**				
BMI	26.1	10.9	21.9	0.67
TMI	14.7	9.9	23.4	0.67
RFM	25.2	6.7	19.0	0.74
**Obesity†**				
	**Cutoff**	**FNR (%)**	**FPR (%)**	**Youden's index**
**Girls 8 to 14 years of age**				
BMI	25.3	4.9	12.7	0.82
TMI	16.3	1.9	13.7	0.84
RFMp	40.6	5.6	9.6	0.85
**Boys 8 to 14 years of age**				
BMI	23.8	4.8	13.9	0.81
TMI	16.0	3.3	11.5	0.85
RFMp	34.5	0.4	10.8	0.89
**Girls 15 to 19 years of age**				
BMI	31.2	4.0	8.0	0.88
TMI	19.4	5.5	7.2	0.87
RFM	41.9	5.1	12.5	0.82
**Boys 15 to 19 years of age**				
BMI	29.5	4.1	11.9	0.84
TMI	16.6	4.3	12.2	0.84
RFM	29.9	7.9	7.6	0.85

FNR, false negative rate (1−sensitivity); FPR, false positive rate (1−specificity).

* Diagnosis based on a DXA-measured whole-body fat percentage ≥85th and <95th percentile.

† Diagnosis based on a DXA-measured whole-body fat percentage of ≥95th percentile.

Supplementary Table 7. Correlation matrix (Pearson's r) between anthropometrics and biomarkers for cardiometabolic disease among children and adolescents 8 to 14 years of age.

	log-Insulin*	log-Glucose	LDLc*	log-HDLc	log-TAG*	log-Total cholesterol	log-(TAG/HDLc)
**Girls (n=507)**							
log-Body mass index (BMI)	0.60†	0.01	0.13§	−0.20§	0.20‡	0.09	0.23‡
log-Waist circumference	0.59†	0.03	0.13§	−0.23‡	0.26†	0.09	0.30†
DXA-whole-body fat percentage	0.57†	0.07	0.14§	−0.19‡	0.26†	0.12§	0.28†
DXA-trunk fat percentage	0.60†	0.05	0.18‡	−0.21‡	0.28†	0.14§	0.30†
RFMp	0.59†	0.01	0.15§	−0.26†	0.27†	0.10	0.31†
Waist-to-height ratio	0.59†	0.04	0.15§	−0.25†	0.26†	0.09	0.30†
**Boys (n=714)**							
log-Body mass index (BMI)	0.68†	0.09	0.15§	−0.49†	0.35†	0.06	0.45†
log-Waist circumference	0.69†	0.09	0.13§	−0.49†	0.40†	0.05	0.49†
DXA-whole-body fat percentage	0.54†	0.05	0.27†	−0.37†	0.39†	0.21†	0.44†
DXA-trunk fat percentage	0.60†	0.04	0.27†	−0.41†	0.43†	0.21†	0.48†
RFMp	0.63†	0.09	0.21†	−0.44†	0.41†	0.14§	0.48†
Waist-to-height ratio	0.65†	0.09	0.20‡	−0.45†	0.41†	0.14§	0.48†

HDLc, high-density lipoprotein cholesterol; LDLc, low-density lipoprotein cholesterol; TAG, triacylglycerol; TAG/HDLc, ratio of TAG to HDLc.

* Measured in the fasting state. †$P<0.001$; ‡$P<0.01$; §$P<0.05$.

Supplementary Table 8. Correlation matrix (Pearson's r) between anthropometrics and biomarkers for cardiometabolic disease among adolescents 15 to 19 years of age.

	log-Insulin*	log-Glucose	LDLc*	log-HDLc	log-TAG*	log-Total cholesterol	log-(TAG/HDLc)
**Girls (n=882)**							
log-Body mass index (BMI)	0.52†	0.16†	0.17†	−0.32†	0.15†	0.06	0.26†
log-Waist circumference	0.48†	0.18†	0.18†	−0.33†	0.19†	0.07	0.29†
DXA-whole-body fat percentage	0.46†	0.15†	0.22†	−0.30†	0.16†	0.11‡	0.26†
DXA-trunk fat percentage	0.50†	0.16†	0.25†	−0.34†	0.19†	0.13§	0.30†
RFM	0.48†	0.17†	0.19†	−0.34†	0.19†	0.07	0.30†
Waist-to-height ratio	0.50†	0.18†	0.18†	−0.32†	0.18†	0.08	0.28†
**Boys (n=1,207)**							
log-Body mass index (BMI)	0.56†	0.14†	0.29†	−0.33†	0.38†	0.29†	0.42†
log-Waist circumference	0.59†	0.15†	0.28†	−0.37†	0.43†	0.28†	0.47†
DXA-whole-body fat percentage	0.59†	0.17†	0.26†	−0.34†	0.38†	0.25†	0.42†
DXA-trunk fat percentage	0.61†	0.16†	0.28†	−0.36†	0.43†	0.28†	0.47†
RFM	0.59†	0.16†	0.29†	−0.34†	0.42†	0.30†	0.45†
Waist-to-height ratio	0.60†	0.15†	0.29†	−0.35†	0.42†	0.29†	0.46†

HDLc, high-density lipoprotein cholesterol; LDLc, low-density lipoprotein cholesterol; TAG, triacylglycerol; TAG/HDLc, ratio of TAG to HDLc.

* Measured in the fasting state. †P<0.001; ‡P<0.05; §P<0.01.

REFERENCE

1. Woolcott, O. O. & Bergman, R. N. Relative fat mass (RFM) as a new estimator of whole-body fat percentage ─ A cross-sectional study in American adult individuals. *Sci Rep* **8**, 10980 (2018).
